# Engineered triple inhibitory receptor resistance improves anti-tumor CAR-T cell performance via CD56

**DOI:** 10.1038/s41467-019-11893-4

**Published:** 2019-09-11

**Authors:** Fan Zou, Lijuan Lu, Jun Liu, Baijin Xia, Wanying Zhang, Qifei Hu, Weiwei Liu, Yiwen Zhang, Yingtong Lin, Shuliang Jing, Mei Huang, Bifen Huang, Bingfeng Liu, Hui Zhang

**Affiliations:** 1 Institute of Human Virology, Zhongshan School of Medicine, Sun Yat-sen University, Guangzhou, Guangdong 510080 China; 2 Key Laboratory of Tropical Disease Control of Ministry of Education, Guangzhou, Guangdong 510080 China; 3 Guangdong Engineering Research Center for Antimicrobial Agent and Immunotechnology, Guangzhou, Guangdong 510080 China; 4 Qianyang Biomedical Research Institute, Guangzhou, Guangdong 510663 China

**Keywords:** Cancer therapy, Immunotherapy, Cytotoxic T cells

## Abstract

The inhibitory receptors PD-1, Tim-3, and Lag-3 are highly expressed on tumor-infiltrating lymphocytes and compromise their antitumor activity. For efficient cancer immunotherapy, it is important to prevent chimeric antigen receptor T (CAR-T)-cell exhaustion. Here we downregulate these three checkpoint receptors simultaneously on CAR-T cells and that show the resulting PTL-CAR-T cells undergo epigenetic modifications and better control tumor growth. Furthermore, we unexpectedly find increased tumor infiltration by PTL-CAR-T cells and their clustering between the living and necrotic tumor tissue. Mechanistically, PTL-CAR-T cells upregulate CD56 (NCAM), which is essential for their effector function. The homophilic interaction between intercellular CD56 molecules correlates with enhanced infiltration of CAR-T cells, increased secretion of interferon-γ, and the prolonged survival of CAR-T cells. Ectopically expressed CD56 promotes CAR-T cell survival and antitumor response. Our findings demonstrate that genetic blockade of three checkpoint inhibitory receptors and the resulting high expression of CD56 on CAR-T cells enhances the inhibition of tumor growth.

## Abstract (web summary)

The inhibitory receptors PD-1, Tim-3 and Lag-3 act as negative feedback regulators of T cell responses. Here the authors improve CAR T cell antitumor efficacy by triple knockdown of these receptors, show it requires CD56, and correlate CD56-mediated homophilic cell interactions with CAR T cell efficacy.

## Introduction

The tumor-infiltrating lymphocytes (TILs) eventually enter an exhausted status and loss their antitumor function. This phenomenon is largely due to various inhibitory receptors receiving negative signals on their surfaces^1,2^. Many inhibitory co-receptors including CTLA-4, PD-1, Tim-3, Lag-3, Tigit, CD160, Vista, and 2B4 have been identified^3–10^, the expression of which are upregulated in different tumor microenvironments^11^. The interaction of PD-1 and corresponding ligand PD-L1 results in the recruitment of Src-homology domain-containing phosphatase 1 (SHP1) and SHP2, which facilitate the dephosphorylation of cytoplasmic domain of CD28 and eventually upregulate BATF expression through a signal cascade^12,13^. The T lymphocytes in the exhausted status fail to kill the target cells and lack the secretion of various cytokines including interferon-γ (IFN-γ), tumor necroptosis factor-α (TNF-α), interleukin-2 (IL-2), etc. They have specific epigenetic and chromatin structural changes^14,15^. The blockage of co-inhibitory receptors and corresponding ligands significantly enhances the antitumor activity of TILs. Recent clinical success of antibody therapy targeting co-inhibitory molecules such as PD-1 or CTLA-4 underscores the therapeutic potential of counteracting immune inhibition^16,17^.

Chimeric antigen receptor T cells (CAR-T cells) have exerted potent antitumor activity. Although they effectively eradicate the leukemic/lymphoma cells and lead to an impressive clinic success, their inhibitory effect on solid tumors is compromised^18^. The combination of CAR-T cells with antibody targeting co-inhibitory molecule, or the expression knockdown or other intrinsic blockades of PD-1 on the CAR-T cells, have exerted an enhanced antitumor activity^19–22^. Recently, more efforts have been made to improve the anti-solid tumor activity, including coexpression of IL-7 and CCL19, heparanase, IL-12, or CCR2b in the CAR-T cells^23–27^. However, their enhancement for antitumor activity in patients remains to be determined. More strategies for improving the anti-solid tumor effect of CAR-T cells are urgently required.

It has been shown that PD-1, Tim-3, and Lag-3 are highly expressed on TILs in various solid tumor types^4,28–31^, as well as on CD8^+^ T cells during chronic viral infections^32,33^. In this study, we simultaneously decrease the expression of PD-1, Tim-3, and Lag-3 in CAR-T cells (PTL-Her2-CAR-T cells) by short hairpin RNA (shRNA) cluster and find that this treatment significantly enhances the anti-Her2^+^-solid tumor effect of Her2-specific CAR-T cells. We find that the infiltration of PTL-Her2-CAR-T cells into the xenograft tumor tissue is significantly increased. Further studies demonstrate that the CD56 molecule is upregulated on these PTL-Her2-CAR-T cells and the homophilic interaction between intercellular CD56 molecules potently enhances the anti-solid tumor activity and prolonged survival.

## Results

### The PTL-Her2-CAR-T cells exerted potent antitumor activity

**Fig. 1:**
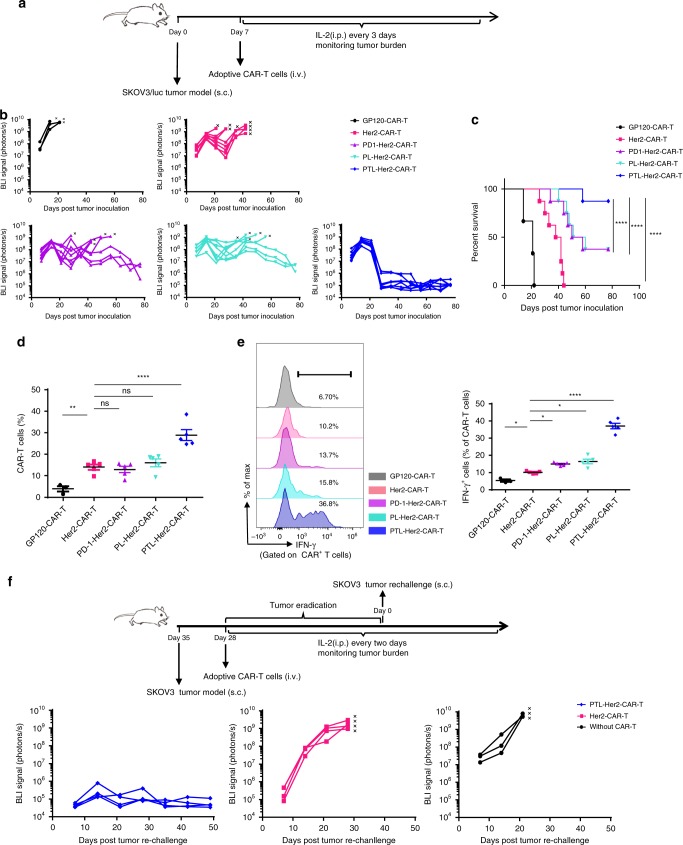
PTL-Her2-CAR T cells exerted a potent antitumor activity and infiltration in tumor tissue. **a** Schematic diagram of a complete animal experiment for **b**, **c**. SKOV3/luc cells were inoculated subcutaneously into NSG mice. Seven days later, various CAR-T cells were infused via tail injection and GP120-CAR-T cells were used as mock CAR-T cells. All the mice were analyzed on the IVIS once a week (*n* = 3, GP120-CAR-T group; *n* = 8, other groups). **b** Tumor burden over time showed bioluminescent signal quantified per mouse. The black cross means death or killing of the debilitated mouse. **c** Kaplan–Meier survival analysis of mice. **d**, **e** SKOV3 cells were inoculated subcutaneously into NSG mice. Seven days later, various CAR-T cells were infused via tail injection (*n* = 3 for GP120-CAR-T group and *n* *=* 5 for other groups). At day 28 after CAR-T cell infusion, tumor tissues were resected from the mice, followed by digestion and FACS analysis. **d** The percentage of infiltrating CAR-T cells in tumor. **e** Left panel shows representative flow cytometry analysis of IFN-γ-positive cells; right panel shows the percentages of IFN-γ-positive cells in tumor-infiltrating CAR-T cells among different groups. **f** Up panel shows schematic diagram of SKOV3/luc tumor re-challenge assay. SKOV3/luc cells were inoculated subcutaneously into the opposite hemisphere of NSG mice. Seven days later, 2 × 10^6^ Her2-CAR-T cells or PTL-Her2-CAR-T cells per mouse, which were double dose than that for the above conventional CAR-T treatment, were infused via tail injection (*n* = 4 for each group). At day 28 after CAR-T cell infusion, long-term surviving mice were re-challenged with a second inoculation of SKOV3/luc cells. The other three NSG mice were inoculated and used as positive control. The black cross means death or killing of the debilitated mice. *P *<* 0.05, **P *<* 0.01, ****P *<* 0.0001. ns, no significant difference (one-way ANOVA with Tukey’s post hoc tests (**d**, **e**); log-rank Mantel–Cox test (**c**)). All data are means ± SEM. Data shown are representative of two independent experiments. The source data underlying **b–f** are provided as a Source Data file

At first, we constructed a Her2-specific CAR in lentiviral vector with specific cytotoxicity to Her2^+^ human ovarian cancer SKOV3 cells (Supplementary Fig. 1), and its combination with one of sh-PD-1, sh-Tim-3, sh-Lag-3, or scramble shRNA (Supplementary Fig. 2a). The shRNA rather than sgRNA-guided CRISPR-CAS9 was used in our study, as the decreased expression rather than complete inactivation of these genes could be better for the survival of T cells in the tumor microenvironment because of their other requisite functions^34–36^. The specific shRNA cluster of PD-1, Tim-3, and Lag-3 led to a significant repression on the mRNAs of their target genes (Supplementary Fig. 2b). After confirming that the Her2-specific CAR-T cells with the downregulation of each inhibitory receptor exerted higher Her2-specific cytotoxicity (Supplementary Fig. 2c, d), we generated the Her2-specific CAR-T cells carrying shRNA cluster for three co-inhibitory molecules, which was thereafter named as PTL-Her2-CAR-T cells. More than 80% knockdown efficiency of all three receptors was found in PTL-Her2-CAR-T cells, when compared with the control Her2-CAR-T cells (Supplementary Fig. 3a). The combined downregulation of three inhibitory receptors simultaneously exerted much more synergistic effect than the downregulation of single or double inhibitory receptors based on the specific cytotoxicity and IFN-γ secretion of CAR-T cells (Supplementary Fig. 3b, c). Furthermore, PTL-Her2-CAR-T cells and the control CAR-T cells were administered separately into SKOV3-bearing NOD-Prkdc^scid^ Il2rg^null^ (NSG) mice via a single intravenous (i.v.) injection (Fig. 1a). A much stronger antitumor activity of PTL-Her2-CAR-T cells was observed (Fig. 1b). Importantly, the survival of mice treated with PTL-Her2-CAR-T cells had significantly improved (Fig. 1c) and nonspecific infiltration was found in the normal tissues of these mice (Supplementary Fig. 4). Similar results were observed when the CD19^+^ Raji-bearing NSG mice were injected with CD19-specific CAR-T cells carrying shRNA cluster for three co-inhibitory molecules (PTL-CD19-CAR-T cells) (Supplementary Fig. 5). Although the sustained downregulation of inhibitory receptors on PTL-CAR-T cells in vivo at different time points (Supplementary Fig. 6), the infiltration and IFN-γ secretion of PTL-Her2-CAR-T cells had significantly elevated in the xenograft tumor tissue (Fig. 1d, e). In addition, to test the in vivo function of persisting long-term PTL-CAR-T cells, we performed a tumor re-challenge experiment on day 28 after which the double-dose CAR-T cells were delivered to ensure the tumor can be efficiently inhibited in the conventional Her2-CAR-T cells treatment group. We found that an effective control of tumor re-challenge occurred in PTL-CAR-T cell-treated group, whereas only a weak control of tumor occurred in Her2-CAR-T cell-treated group (Fig. 1f).

It has been reported that the efficient induction of central memory phenotype with high expression of CD62L and CD127 is essential for the persistent control of tumors in immunotherapy^37,38^. We then examined the possible memory phenotypes of CAR-T cells in the xenograft-bearing mice, which survived for 60 days after adoptive transferred. Her2-CAR-T cells and the control GP120-CAR-T cells were also introduced in our experiment. Human CD8^+^ T cells were highly accumulated in the spleen of mice treated with PTL-Her2-CAR-T cells compared with those in the control CAR-T cells (Supplementary Fig. 7a). We further identified that CD62L^+^ CD127^+^ CAR-T cells were significantly higher in the spleen of mice treated with PTL-Her2-CAR-T cells than that treated with conventional CAR-T cells (Supplementary Fig. 7b).

**Fig. 2:**
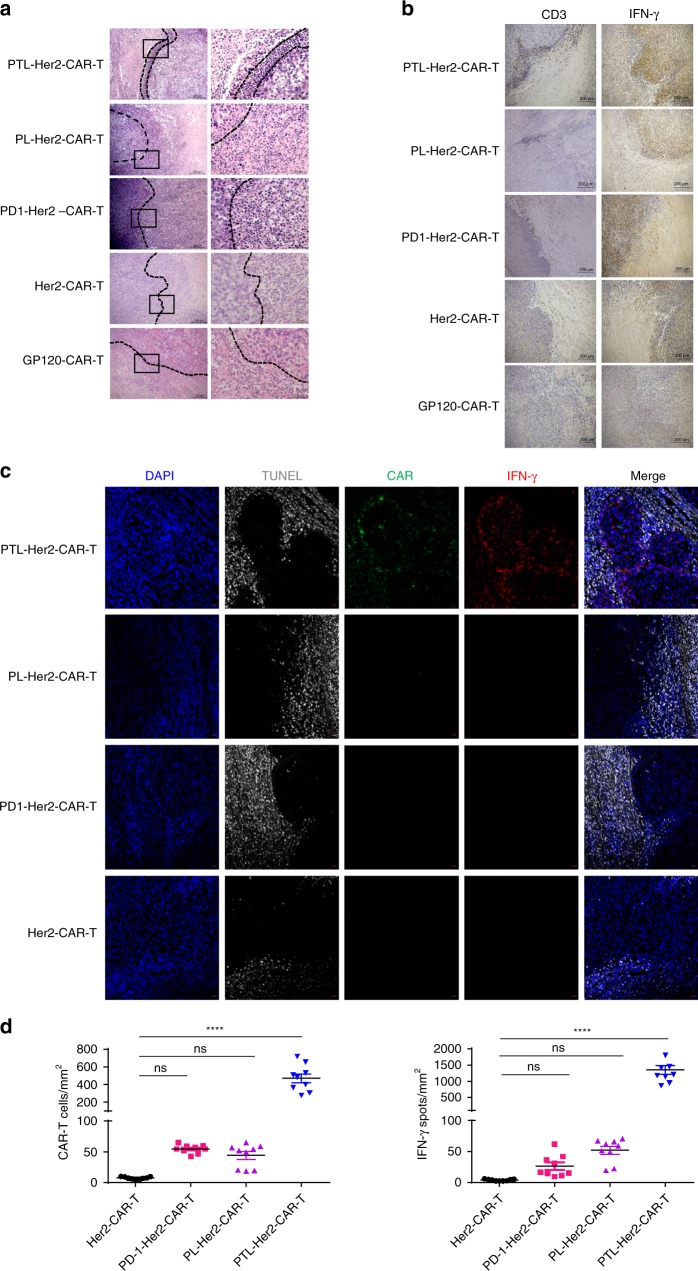
The PTL-Her2-CAR T cells exerted enhanced infiltration in the tumor tissue. SKOV3-bearing mice cells were treated with various CAR-T cells. At day 28 after CAR-T cell infusion, mice were killed and tumor tissues were resected from the mice and stained with hematoxylin and eosin (H&E), immunohistochemistry (IHC), or immunofluorescence staining. **a** The H&E staining of tumor tissue sections. Right panel shows higher magnification from the box in the left respectively (left, scale bar 200 µm; right, scale bar 50 µm). **b** Anti-human CD3 antibody and anti-human IFN-γ antibody were used for primary staining in IHC analysis (scale bar, 200 µm). **c** Immunofluorescence staining of tumor frozen tissue sections. CAR-T cells were stained with anti-mouse IgG H&L (Alexa Fluor 488) and IFN-γ was stained with anti-human IFN-γ (Alexa Fluor 647). Apoptosis-undergoing cells were stained by TUNEL assay (Roche TMR-RED) and nuclei were stained with DAPI (blue). Scale bar, 20 µm. Pictures shown are representative of two independent experiments. **d** Left panel, the percentages of CAR^+^ T cells per mm^2^ found in different CAR-T treatment groups; right panel, the percentages of IFN-γ^+^ spots per mm^2^ found in different groups. At least three separate fields from each mice and three mice from each group were calculated. ****P *<* 0.0001. ns, no significant difference (one-way ANOVA with Tukey’s post hoc tests (**d**)). All data are means ± SEM. Data shown are representative of two independent experiments. The source data underlying **b–f** are provided as a Source Data file

Histological sections of xenograft tumor tissue from mice treated with PTL-Her2-CAR-T cells also showed enhanced CAR-T cell infiltration and an extensive IFN-γ release in this margin area (Fig. 2a, b). Immunofluorescent staining in all engrafted tumor tissue sections further confirmed that PTL-Her2-CAR-T cells significantly increased infiltration compare with other CAR-T cell treatment group. Around and within these CAR-T cells, extensive IFN-γ release was also observed (Fig. 2c, d and Supplementary Fig. 8). Given the important role of INF-γ in inducing apoptosis of tumor cells^39,40^, it is reasonable to assume that the apoptosis-undergoing tumor tissue nearby could be the victims of the CAR-T cells gathering within the living tumor tissue.

### CD56 mediated the homophilic interaction of CAR-T cells

**Fig. 3:**
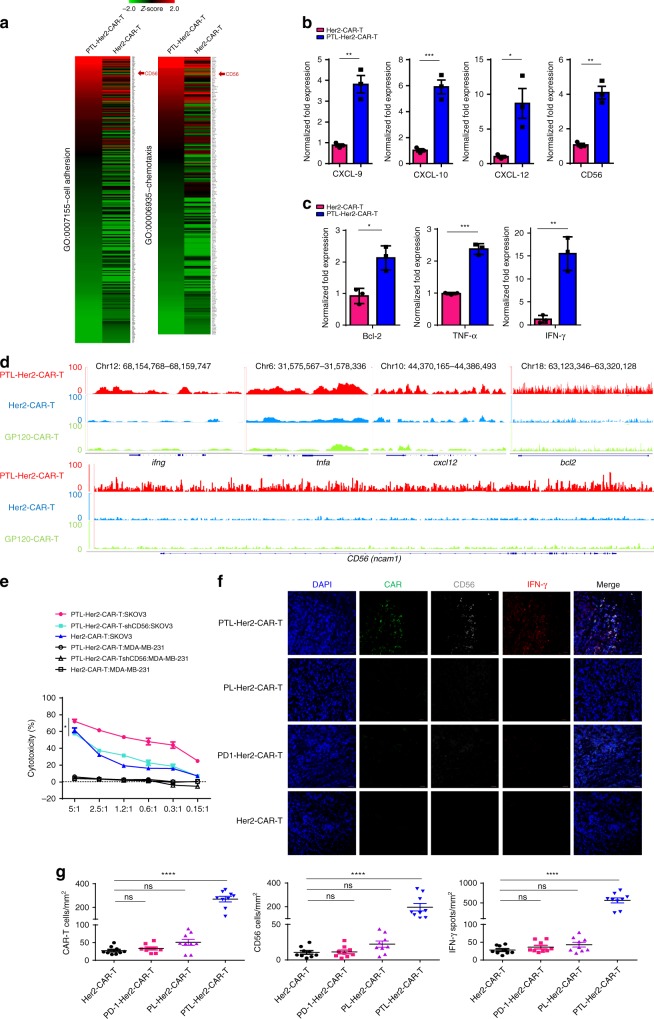
The increased CD56 is the key factor for gathering of PTL-Her2-CAR-T cells. **a**–**d**, **f**, **g** SKOV3-bearing NSG mice were injected with various CAR-T cells via tail. At 28 days after CAR-T-cell infusion, tumor tissues were resected. Each tissue was divided into two parts. One part was digested and tumor-infiltrating CAR-T cells were separated for RNA-seq (**a**), qRT–PCR (**b**, **c**), ATAC-seq (**d**), and another for immunofluorescence staining (**f**). **a** Heatmaps showing the *z*-score normalized expression of the differentially expressed genes involved in the cell adhesion and chemotaxis, which were significantly enriched with Gene Ontology (GO) biology process terms with *P*-value < 0.001. RNA expression levels are indicated with a red/green scale for high and low expression levels, respectively. **b**, **c** Relative mRNA levels of genes were verified by qRT–PCR. **d** Genome alignment tracks of the normalized ATAC-seq data showing the open chromatin for the genes *ifng*, *tnfa*, and *cxcl12*, *bcl2*, and *CD56* in various CAR-T cells. **e** Cytotoxic activities of PTL-Her2-CAR-T (*n* = 3, 3 healthy donors) cells with CD56 knockdown on SKOV3 cells and MDA-MB-231 (negative control). **f** Immunofluorescence staining of tumor frozen tissue sections. CAR-T cells were stained with anti-mouse IgG H&L (green). CD56 was stained with anti-human CD56 (white) and IFN-γ was stained with anti-human IFN-γ (red). Nuclei were stained with DAPI (blue). Scale bar, 20 µm. **g** Left panel, the percentages of CAR^+^ T cells per mm^2^ found in different CAR-T treatment groups; middle panel, the percentages of CD56^+^ cells per mm^2^ in different groups; right panel, the percentages of IFN-γ^+^ spots per mm^2^ in different groups. At least three separate fields from each mouse and three mice from each group were calculated. *P *<* 0.05, **P *<* 0.01, ***P *<* 0.001, ****P *<* 0.0001. ns, no significant difference (one-way ANOVA with Tukey’s post hoc tests (**e**, **g**); two-tailed Student’s *t*-test (**b, c**)). All data are means ± SEM. **b**, **c**, **e** Data shown are representative data from three independent experiments and (**a**, **d**, **f**) from two independent experiments. The source data underlying **b**, **c**, **e** and **g** are provided as a Source Data file

Based on the results observed above, we hypothesized that cell-to-cell adhesion or chemotaxis to each other of PTL-Her2-CAR-T cells may be the key contributors of increased infiltration. To this end, we compared the gene expression profiles between the PTL-Her2-CAR-T cells and Her2-CAR-T cells isolated from tumor tissues, respectively. Gene Ontology (GO) enrichment analysis revealed a significant increase of some chemokine family members such as *cxcl9*, *cxcl10*, and *cxcl12*, and notably a cell adhesion factor, *CD56* (*NCAM*, *neural cell adhesion molecule*) in the PTL-Her2-CAR-T cells isolated from tumor tissues (Fig. 3a), which were validated by quantitative real-time PCR (qRT–PCR) analysis (Fig. 3b). Moreover, the PTL-Her2-CAR-T cells also showed increased expression of multiple genes encoding effector molecules, such as IFN-γ, TNF-α, and Bcl-2 (Fig. 3c). Through ATAC-seq technique, we found that some gene loci of upregulated gene expression in PTL-CAR-T cells from xenograft-derived tumor, incliuding *ifng* (Chr12: 68,154,768-68,159,747), *tnfa* (Chr6: 31,575,567-31,578,336), and *cxcl12* (Chr10: 44,370,165-44,386,493), *bcl2* (Chr18: 63,123,346-63,320,128), and *CD56* (Chr11: 112,961,247-113,278,436), exhibited much higher peaks, indicating that the chromatin accessibility of many functional genes had significantly changed (Fig. 3d).

**Fig. 4:**
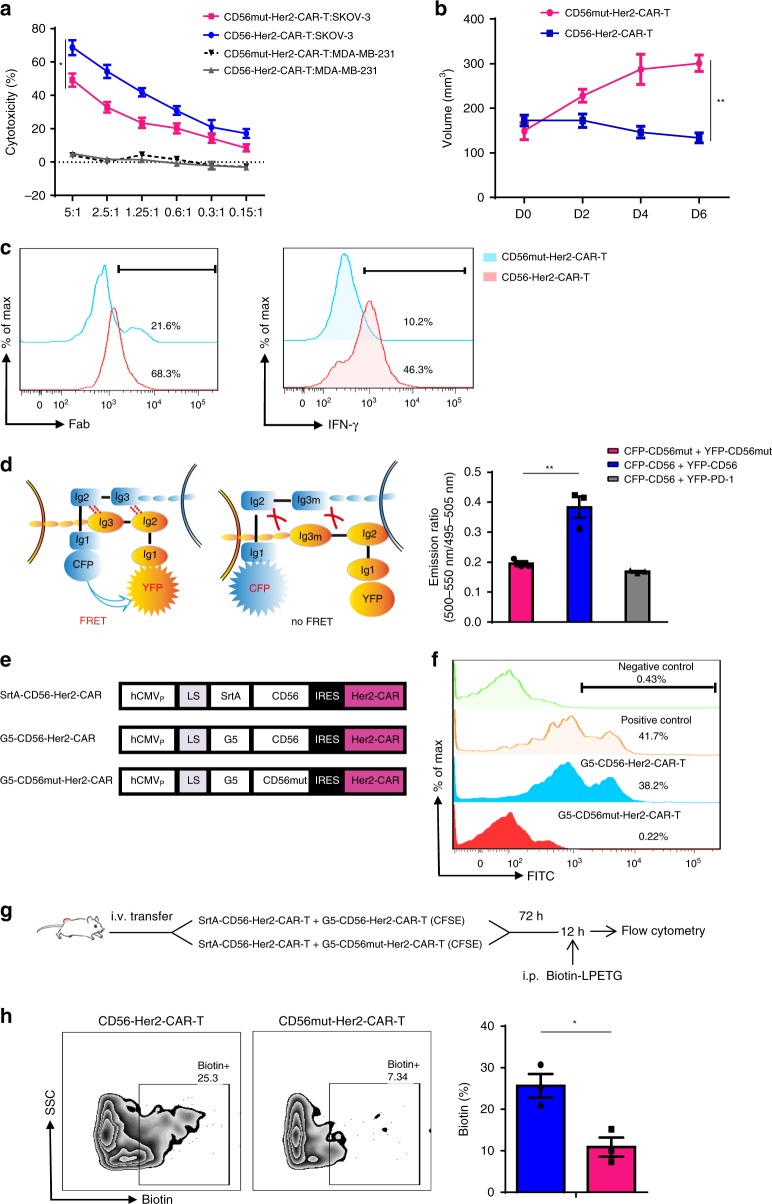
The homophilic interaction of CD56 mediated the interaction of CAR-T cells. **a** The effect of CD56 on the cytotoxic activity of Her2-CAR-T cell-targeted SKOV3 cells were detected by LDH release assay and MDA-MB-231 cells were used as negative control (*n* = 3, 3 healthy donors). **b**, **c** SKOV3-bearing NSG mice were infused via tail with CD56-Her2-CAR-T cells or CD56mut-Her2-CAR-T cells, respectively. Tumor was monitored and 6 days later tumor tissues were resected and digested for FACS. **b** volume of mice (*n* = 6, CD56mut-Her2-CAR-T group; *n* *=* 4, CD56-Her2-CAR-T group). **c** Left panel, representative flow cytometry analysis of tumor-infiltrating CAR-T cells by detecting the scFv fragment of CAR moiety with anti-Fab antibody. Right panel, the percentages of IFN-γ-positive cells in tumor-infiltrating CAR-T cells of different groups. **d** Left panel, schematic diagram of FRET assay between intercellular CD56. Right panel, the increased FRET between the CFP (cyan fluorescent protein)-CD56-expressing CD8^+^ T cells and YFP (yellow fluorescent protein)-CD56-expressing CD8^+^ T cells (*n* = 3, 3 healthy donors). CFP-CD56-expressing CD8^+^ T cells mixed with YFP-PD-1-expressing CD8^+^ T cells were measured as negative control. **e** Construction for lentiviral vectors carrying Her2-CAR-T moiety and SrtA-CD56, G5-CD56, or G5-CD56mut, respectively. **f** Histograms show conjugated AlexaFluor488 in G5-CD56-Her2-CAR T cells or G5-CD56mut-Her2-CAR T cells (indicating intercellular transfer) in vitro. SrtA-PD-1 cells with G5-PD-L1 cells were used as positive control and SrtA-PD-1 cells with G5-CD56-Her2-CAR T cells as negative control. Data are representative of three independent experiments. **g** The procedures for LIPSTIC assay in vivo. **h** Left panel shows the biotin labeling of G5-CD56-Her2-CAR T cells or G5-CD56mut-Her2-CAR T cells (CFSE-labeled) in tumor by flow cytometric analysis. Right panel shows the percentage of biotin^+^ G5-Her2-CAR T cells gated as in the left panel (*n* *=* 3 per group). *P *<* 0.05, **P *<* 0.01 (one-way ANOVA with Tukey’s post hoc tests (**a**, **d**); two-tailed Student’s *t*-test (**b**, **h**)). All data are means ± SEM. (**a**, **d**, **f**) Data shown are representative data from three independent experiments and (**b**, **c**, **h**) from two independent experiments. The source data underlying **a**, **b**, **d** and **h** are provided as a Source Data file

To identify the key factors contributing to the increased antitumor activity of PTL-Her2-CAR-T cells, we further knocked down the upregulated factors related to cell-to-cell adhesion or chemotaxis, respectively. Only PTL-Her2-CAR-T cells with CD56 downregulation showed a significantly reduced cytotoxicity activity in vitro (Fig. 3e and Supplementary Fig. 9), indicating that the increased CD56 played a key role in enhancing antitumor activity. Furthermore, we performed immunofluorescence by staining human CD56, CD3, and IFN-γ in xenograft-derived tissue section and found that CD56^+^ cells strongly colocalized with the infiltrated CD3^+^-PTL-Her2-CAR-T cells and the IFN-γ secretion (Fig. 3f, g and Supplementary Fig. 10). Similar upregulation of CD56 was also observed in PTL-CD19-CAR-T cells, which infiltrated into CD19^+^ Raji tumor tissues (Supplementary Fig. 11). Nevertheless, although the treatment with Her2-CAR-T cells plus anti-PD-1 blocking antibody also exerted an enhanced antitumor effect, the expression of CD56 molecule on Her2-CAR-T cells was not significantly changed (Supplementary Fig. 12). Together, these results suggested that the triple downregulations of PD-1, Tim-3, and Lag-3 lead to the upregulation of CD56 and the enhanced infiltration of PTL-Her2-CAR-T cells in tumor tissue could be mediated by CD56. The homophilic interaction of CD56 has been well investigated in neural system^41,42^. The Ig1 and Ig2 modules of CD56 mediate the dimerization of CD56 molecules situated on the same cell surface (*cis* interactions), whereas the Ig3 module mediates the interactions between CD56 molecules expressed on the surface of opposing cells (*trans* interactions) through simultaneously binding to the Ig1 and Ig2 modules^42,43^. We hypothesized that the intercellular homophilic interaction of CD56 could contribute to the interaction of PTL-Her2-CAR-T cells. As Lys303 and Phe305 from Ig3 domain of CD56 mediate the *trans* interactions of CD56 on the surface of opposing cells^41,42^, we inserted the genes of *CD56* or *CD56* with mutations K303A and F305A, which thereafter is named as CD56mut, into Her2-CAR-expressing vector. The CD56 overexpression in Her2-CAR-T cells displayed increased antitumor activity in vitro, whereas CD56mut did not (Fig. 4a). Moreover, in the adoptively transferred to SKOV3-bearing NSG mice experiment, the CD56-overexpressing Her2-CAR-T cells displayed significantly enhanced antitumor ability, whereas the overexpression of CD56mut did not (Fig. 4b). In addition, the infiltration and IFN-γ secretion of CD56-expressing CAR-T cells in the tumor tissues were significantly elevated in comparison with that of CD56mut-expressing CAR-T cells (Fig. 4c), which indicated that the homophilic interaction of CD56 was important for the infiltration, survival, and antitumor activity of Her2-CAR-T cells.

To search for more evidence of CD56 homophilic interaction between the CD56^+^ CAR-T cells, we generated CFP-CD56- and YFP-CD56-expressing vectors and transduced them into CD8^+^ T cells separately. We found that the emission ratio of fluorescence resonance energy transfer (FRET) between the CFP-CD56 and YFP-CD56 significantly increased, indicating that the homophilic interaction of CD56 occurred intercellularly (Fig. 4d). Moreover, we introduced the “Labelling Immune partnerships by SorTagging Intercellular contacts” (LIPSTIC) system to measure the CD56-CD56 homophilic interactions enzymatically^44^. The bicistronic vectors co-expressing the Her2-CAR with G5-CD56 or SrtA-CD56 were transduced into CD8^+^ T cells, respectively, and CD56-CD56 interaction was confirmed by LIPSTIC labeling in cell culture (Fig. 4e, f). Then we adoptively co-transferred Her2-CAR-T cells carrying G5-CD56 or SrtA-CD56 into SKOV3-bearing NSG mice, followed by LIPSTIC labeling at 72 h later (Fig. 4g), and found that LIPSTIC labeling was on the tumor-infiltrated G5-CD56-I-CAR T cells, while not on the G5-CD56mut-I-CAR T cells, which directly confirmed the CD56-CD56 intermolecular interaction between CAR-T cells in vivo (Fig. 4h).

### Homophilic interaction of CD56 enhanced antitumor activity

**Fig. 5:**
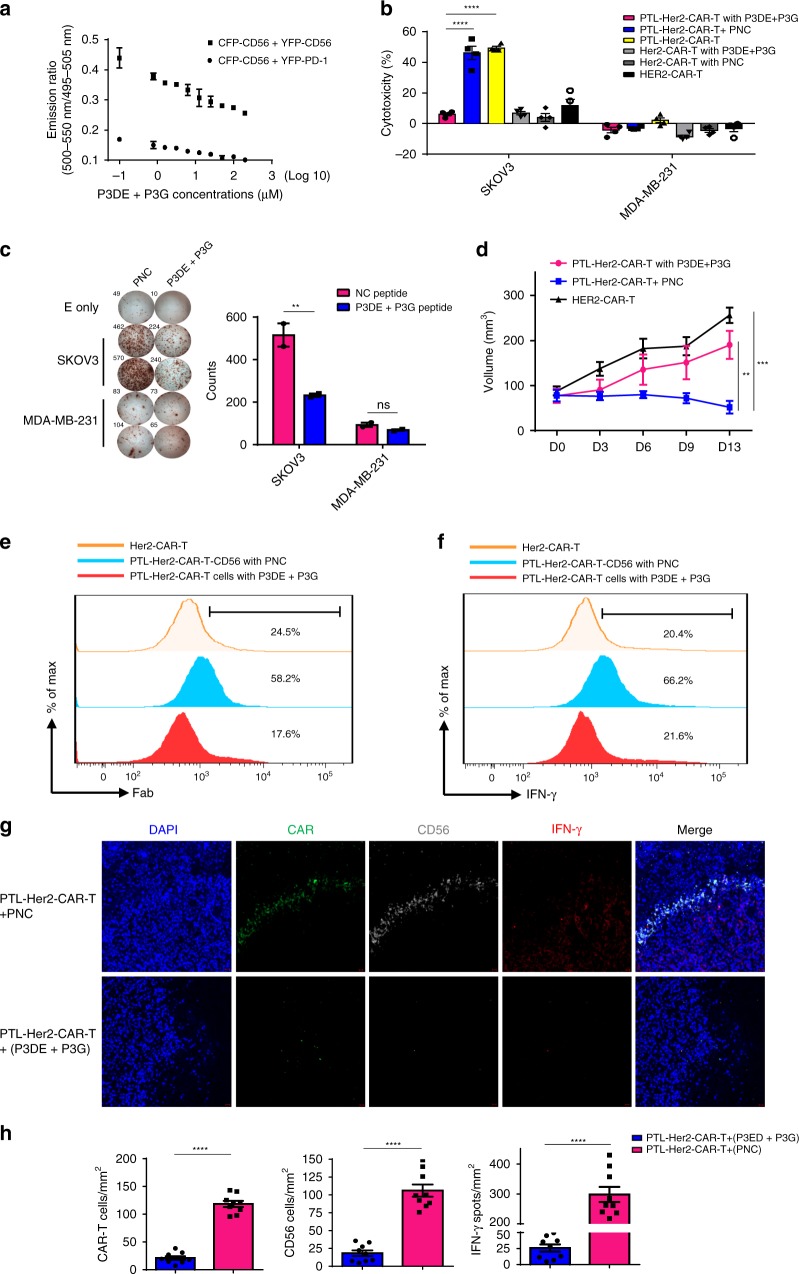
Homophilic interaction of CD56 enhanced the antitumor activity of CAR-T cells. **a** Graph shows the emission ratio of FRET between the CFP-CD56 and YFP-CD56. Data were plotted as 500~550 nm/495~505 nm ratios vs. the concentration of P3DE + P3G peptides. **b**, **c** The effect of the P3DE + P3G on the function of PTL-Her2-CAR-T cells targeting SKOV3 cells were detected by cytotoxic activity (*n* = 4, 4 healthy donors) (**b**) and IFN-γ ELISpot assay (*n* = 2, 2 healthy donors) (**c**). All peptides were used at a concentration of 200 μg ml^−1^. **d**–**h** SKOV3-bearing NSG mice were infused via tail with various CAR-T cells. The mice receiving PTL-Her2-CAR-T cells infusion were divided into two groups, with P3DE + P3G or PNC peptide, respectively. Thirteen days later, mice were killed and tumor tissues were digested for FACS. **d** Volume of mice (*n* = 4–5 mice for each group). **e** The representative flow cytometry analysis of CAR-T cells by detecting the scFv fragment of CAR moiety with anti-Fab antibody. **f** The representative flow cytometry analysis of IFN-γ-positive cells in tumor-infiltrating CAR-T cells of different groups. **g** Immunofluorescence staining of the frozen tissue sections of xenograft tumors treated with PTL-Her2-CAR-T cells, in the presence of P3DE + P3G peptides or PNC. CAR-T cells were stained with anti-mouse IgG H&L (green). CD56 was stained with anti-human CD56 (white), IFN-γ was stained with anti-human IFN-γ (red), and nuclei were stained with DAPI (blue). Scale bar, 20 µm. **h** Left panel, the percentages of CAR^+^ T cells per mm^2^ found in different CAR-T treatment groups; middle panel, the percentages of CD56^+^ cells per mm^2^ in different groups; right panel, the percentages of IFN-γ^+^ spots per mm^2^ in different groups. At least three separate fields from each mice and three mice from each group were calculated. **P *<* 0.01, ***P < 0.001, ****P < 0.0001. ns, no significant difference (one-way ANOVA with Tukey’s post hoc tests (**b**, **d**); two-tailed Student’s *t*-test (**c**, **h**)). All data are means ± SEM. The source data underlying **a–d** and **h** are provided as a Source Data file

It was reported that homophilic interaction of CD56 can be effectively blocked by P3DE or P3G peptides in neural cells^42^. We therefore examined the effect of these synthesized peptides upon the homophilic interaction of endogenous CD56 and subsequently the activity of PTL-Her2-CAR-T cells. The FRET assay indicated that the combined P3DE and P3G treatment inhibited the CD56-CD56 interaction in CD8^+^ T cells in a dose-dependent manner (Fig. 5a). The specific cytotoxicity of PTL-Her2-CAR-T cells was reduced from 46.18% to 6.19% after treatment with these peptides rather than the control P3B peptide (Fig. 5b). Accordingly, the levels of IFN-γ secretion were also significantly compromised (Fig. 5c). Furthermore, the PTL-Her2-CAR-T cells were infused in the SKOV3-bearing NSG mice treated with either blocking peptides (P3DE + P3G) or control peptide via a single i.v. injection. We found that the blocking peptides significantly impaired the antitumor infiltration as well as IFN-γ secretion potency of PTL-Her2-CAR-T cells (Fig. 5d–h and Supplementary Fig. 13).

### CD56 enhanced anti-apoptosis ability of PTL-Her2-CAR-T cells

**Fig. 6:**
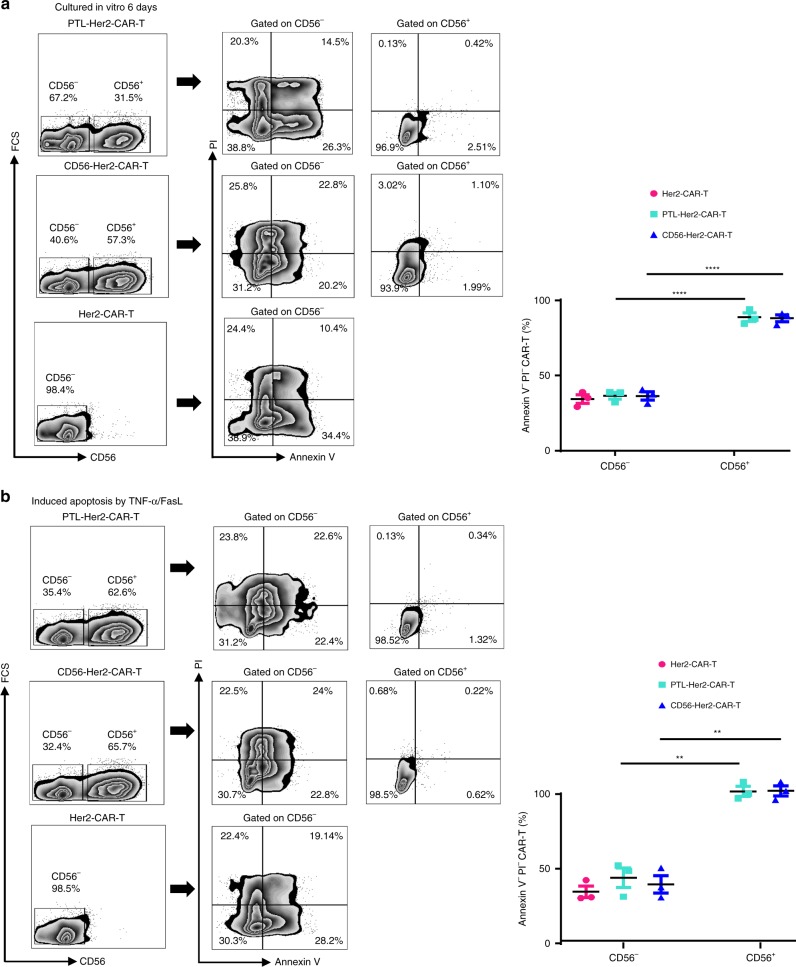
CD56 enhanced anti-apoptosis capability of CAR-T cells. **a** Left panel, the representative flow plots of apoptotic cell proportion in Her2-CAR-T, PTL-Her2-CAR-T, and CD56-Her2-CAR-T cells after culture for 6 days with cytokine deprivation. Right panel, the percentage of Annexin V^−^ and PI^−^ cells gated as in left panel. **b** Left panel, the representative flow plots of apoptotic cell proportion induced by TNF-α and FasL (10 ng ml^−1^) in various CAR-T cells. Right panel, the percentage of Annexin V^−^ and PI^−^ cells gated as in the left panel. **P < 0.01, ****P < 0.0001 (two-tailed Student’s *t*-test). All presented data summarizes mean ± SEM. Data shown are representative data from three independent experiments. The source data underlying **a**, **b** are provided as a Source Data file

As Bcl-2 expression is high in PTL-Her2-CAR-T cells, we measured the anti-apoptosis capability of PTL-Her2-CAR-T or Her2-CAR-T cells carrying exogenous CD56 and found that the percentage of apoptotic cells of PTL-Her2-CAR-T cells and Her2-CAR-T cells carrying exogenous CD56 was much lower than that of conventional Her2-CAR-T cells after culturing for 6 days in the deprivation of any extra cytokines (Fig. 6a). Furthermore, the apoptosis induced by TNF-α and FasL (10 ng ml^−1^) in PTL-Her2-CAR-T cells and Her2-CAR-T cells carrying exogenous CD56 was also significantly lower than that in Her2-CAR-T cells (Fig. 6b). These data suggested that CD56 expression enhanced the anti-apoptosis capability of CAR-T cells.

## Discussion

CD56, also known as NCAM (NCAM1, NCAM-140), play a key role in neuronal growth, development, regeneration, synapse formation, and maintenance^41–43^. Besides, CD56 is a typical cell-surface marker of natural killer (NK) cells. More studies have shown that CD56 is also detected on other lymphoid cells, including γδT cells and activated CD8^+^ T cells, as well as on dendritic cells^45–47^. CD56 is often associated with the activation or cytotoxicity in immune cells^48–51^. It has been reported that the human CD56-positive cytotoxic lymphocytes were more activated and exerted more potent antitumor effect^52^. The human CD56^high^ NK cells are capable to generate more cytokines such as IFN-γ, TNF-α, etc.^53^. Unfortunately, as the CD56 expression in NK cells of mice is quite low, it is remaining unknown why the high expression of CD56 is associated with more activation and the high secretion of IFN-γ and TNF-α. Although an in vitro study indicated that the interaction between CD56^+^ NK cells and CD56^+^ stromal cells could contribute to the motility of NK cells, the molecular mechanism remains to be identified^54^.

Through investigating the mechanism underlying the increased infiltration and antitumor activity of CAR-T cells in the tumor tissue, we demonstrated the significant amount of CD56 occurs on the CAR-T cells after the simultaneous decreased expression of PD-1, Tim-3, and Lag-3. This triple downregulation, rather than single decreased expression of PD-1, cause the epigenetic modifications in CAR-T cells and increased the chromatin accessibility of *CD56* gene, as well as some important genes including IFN-γ, TNF-α, and Bcl-2, and subsequently enhanced their transcriptional expression. Besides, several chemokines including CXCL9, CXCL10, and CXCL12 were upregulated by genome-wide transcriptional profiling of tumor-infiltrated PTL-CAR-T cells. However, in a preliminary in vitro test, we did not find any enhancement migration for PTL-CAR-T cells (data not shown). Nevertheless, it is still possible that these chemokines could play a role in attracting each other for PTL-CAR-T cells within tumor tissue and contribute to the activity of PTL-CAR-T cells.

Overexpression of CD56 in CAR-T cells also exerted a potent antitumor effect. Moreover, the blockage of intercellular CD56 homophilic interaction, either by blocking peptides or CD56 mutation on the interface for homophilic interaction, significantly decreased the antitumor effect. Therefore, by comparing the antitumor effect with or without CD56 expression, and the additional antitumor effect, IFN-γ secretion, resistance to apoptosis, and infiltration or survival of CAR-T cells within tumor tissue, we uncovered the importance of homophilic interaction of CD56. This homophilic interaction could benefit the tumor-killing effect by (1) leading to a much higher local concentration of IFN-γ, which impairs the local angiogenesis and consequently induces the tumor cell death^39,40^; (2) passing the survival signal through CD56 molecule to each other, which could result in the CAR-T or other CD56^+^ immune cells more resistant to the local harsh environment; (3) synchronizing other unknown functions of CD56^+^ immune cells through possible signal pathways such as Fyn/focal adhesion kinase/ERK2, Ras–mitogen-activated protein kinase, phospholipase Cγ, spectrin/CaMKII, spectrin/PKCβ, PAK1, RPTPa, or TrkB, as the homophilic interaction of NCAM (CD56) functions in neural tissue^43,55,56^.

With an unusual approach, we unexpectedly identified the effect of CD56 high expression in immune cells. More investigations are required for further uncovering the mechanisms of how the simultaneous knockdown of PD-1, Tim-3, and Lag-3 leads to the activation of *CD56* gene, and how homophilic interaction of intercellular CD56 molecule could directly enhance the secretion of IFN-γ and the resistance to apoptosis. Furthermore, the manipulation of the CD56 expression, as we described in this work, could be a particularly suitable therapeutic strategy for eradicating various solid tumors and controlling many other diseases.

## Methods

### Cell lines

HEK293T, SKOV3, and MDA-MB-231 cell lines were obtained from ATCC and cultured in Dulbecco’s modified Eagle medium (Gibco, Invitrogen, Carlsbad, CA) supplemented with 10% fetal bovine serum (FBS) (Gibco, Invitrogen, Carlsbad, CA). SKOV3/ luc/RFP cells were established by the infection of SKOV3 with recombinant retroviruses carrying luciferase-IRES-RFP moiety, followed by sorting RFP^high^ cells. Raji cells were also obtained from ATCC and cultured in RPMI 1640 (Gibco, Invitrogen, Carlsbad, CA) supplemented with 10% FBS (Gibco, Invitrogen, Carlsbad, CA). All cell culture media contained 100 U ml^−1^ penicillin and 100 μg ml^−1^ streptomycin. Cells were maintained in a humidified atmosphere containing 5% CO_2_ at 37 °C. As the routine cell lines highly expressed Her2 or CD19, respectively, SKOV3 and Raji cell lines were used for tumor model of ovarian cancer or lymphoma. Cell lines were confirmed for surface expression of target antigens and routinely tested for excluding mycoplasma contamination.

### Isolation and culture of primary human T lymphocytes

Peripheral blood mononuclear cells (PBMCs) were derived from samples obtained from healthy volunteers from anonymous buffy coats of healthy donors (Guangzhou Blood Center, Guangzhou) by Ficoll-Hypaque gradient separation. Primary human CD8^+^ T cells were negatively purified with magnetic beads to a purity of >98% from PBMCs with enrichment set DM (BD-IMag^TM^). T lymphocytes were activated by anti-CD3 (R&D Systems) and anti-CD28 at (R&D Systems) antibodies (Abs) 1 μg ml^−1^ and infected in retronectin-coated plates (Takara Bio, Inc., Shiga, Japan). The transduced T cells were expanded in the conditioned medium containing 90% RPMI 1640 (Gibco, Invitrogen, Carlsbad, CA) supplemented with 10% FBS (Gibco, Invitrogen, Carlsbad, CA), 0.1 mM non-essential amino acids (Gibco, Invitrogen, Carlsbad, CA), 2 mM GlutaMAX (Gibco, Invitrogen, Carlsbad, CA), and 0.05 mM 2-mercaptoethanol at an initial concentration of 1 × 10^6^ cells per ml. Cells were fed twice a week with recombinant IL-2 (10 ng ml^−1^) (R&D Systems).

### Construction of CAR-encoding lentiviral vector

The anti-Her2 scFv was derived from mAb FRP5^57^ and anti-CD19 scFv was derived from mAb FMC63^58^. As shown in Supplementary Fig. 1a, the scFv region was fused with the endodomains of CD28 (nucleotides 460–660; GenBank accession number NM_006139.3), CD137 (nucleotides 640–765; NM_001561.5), and CD3ζ (nucleotides 160–492; NM_198053.2) by overlapping PCR. GP120-CAR, specific targeting gp120 antigen of HIV-1^59^, was used as mock CAR-T cells. Lentiviral vectors carrying a Her2-specific CAR moiety and shRNA derived from the miR-106b cluster were shown in Supplementary Fig. 2a. Sequences of siRNA were listed in Supplementary Table 1.

### Transduction of recombinant lentiviral particles

For the production of pseudotyped lentiviral supernatant, the day before transduction, HEK293T cells were seeded at 8 × 10^6^ cells per 100 mm dish. Twenty-four hours later, the pseudoviruses were generated by co-transfecting HEK293T cells with plasmids encoding various CAR moieties (13.5 μg), pMD.2 G encoding VSV-G envelope (7.5 μg), and a packaging vector psPAX2 (16.5 μg) using phosphate transfection system following the manufacturer’s instructions. Supernatants were collected after 48 h and filtered through a 0.45 μm membrane to remove cell debris. Pseudoviruses were concentrated by ultracentrifugation (Optima XE-100, Beckmann) at 827,000 × *g* for 2 h at 4 °C. Then the activated CD8^+^ T lymphocytes were transduced with lentiviral supernatants using retronectin-coated plates, with polybrene (TR-1003-G, Sigma) at 8 μg ml^−1^, followed by centrifugation for 90 min at 350 × *g* then incubated at 37 °C. Twelve hours later, the recombinant viruses were removed and T cells were expanded in the conditioned medium as described above. The genetically modified T cells were maintained in complete T-cell medium in the presence of IL-2 (fed twice a week, 10 ng ml^−1^) and used for functional assay 14 days after transduction.

### RNA isolation and qRT–PCR

Total RNA was isolated with TRIZOL reagent (Life Technologies) and served as the template for preparing cDNA using a PrimeScript reverse transcription reagent kit (TaKaRa) according to the manufacturer’s instructions. Primers for real-time qRT–PCR were listed in Supplementary Table 2. Quantitative PCR was performed with the SYBR Premix ExTaq Kit (TaKaRa) on a CFX96 Real-Time System (Bio-Rad). Human glyceraldehyde-3-phosphate dehydrogenase was measured as an endogenous control.

### Flow cytometry

CAR moiety was labeled with a donkey anti-mouse Fab (Abcam, 150109). For T-cell phenotyping, the following antibodies were used: mouse anti-human PE-CD8 (Clone HIT8a, 12-0089-42), APC-CD56 (Clone TULY56, 17-0566-42) from eBiosciences; mouse anti-human APC-CD279 (Clone EH12.2H7, 329908), FITC-CD223 (Clone 11C3C65, 369308), Brilliant Violet 421-CD127 (Clone A019D5, 351310), PE/cy7-CD62L (Clone DREG-56, 304822), PE-biotin (Clone 1D4-C5, 409003) from Biolegend; and mouse anti-human Brilliant Violet 421-Tim-3 (Clone 7D3 (RUO), 565562) from BD Horizon. For intracellular staining, T cells were fixed and permeabilized using BD Cytofix/Cytoperm kit as the recommendation of the manufacturer. Anti-human CD8-PE (Clone HIT8a, eBiosciences, 12-0089-42) and donkey anti-mouse Fab (Polyclonal, Abcam, ab150109) were used for extracellular staining, and anti-human IFN-γ-eFluor 450 (Clone 4 S.B3, 85-48-7319-42, eBiosciences) was used for intracellular staining. Stained samples were acquired on a BD FACSAria and analyzed with FlowJo software (Tree Star, Ashland, OR).

### Enzyme-linked immunosorbent assay

Various CAR-T cells were co-cultured with target cells at 5:1 (E:T ratio) in 96-well plates (V bottom). Supernatants were collected after 16 h and cytokine release was analyzed using TNF-α- (Neobioscience) and IL-2- (MultiSciences) specific enzyme-linked immunosorbent assay kit according to the manufacturer’s instructions.

### Enzyme-linked immunosorbent spot

For enzyme-linked immunosorbent spot (ELISpot) assay, CAR-T cells were mixed with target cells (10^4^ cells) at effector-to-target ratios (E:T = 5:1) and then added to the anti-gamma IFN-γ antibody-precoated plates from the human IFN-γ ELISpot assay kit (DKW22-1000-096s; Dakewei), along with a negative control (effector CD8^+^ T cells alone) or positive control (phytohemagglutinin stimulation). Plates were incubated for 16–20 h in a humidified atmosphere containing 5% CO_2_ at 37 °C. The ELISpot assays were then performed according to the manufacturer’s instructions. The plates were scanned by the S6 ultra immunoscan reader (Cellular Technology Ltd) and the number of IFN-γ-positive T cells was calculated by ImmunoSpot software (Version 5.1.34) (Cellular Technology Ltd).

### Cytotoxicity assay

The ability of T cells to kill tumor target cells was measured by lactate dehydrogenase (LDH) release assay. Briefly, CAR-T cells were co-cultured with target cells at different ratios (from 5:1 to 0.15:1) for 24 h in a 96-well plate (V bottom). Then LDH release was measured by the CytoTox96 non-radioactive cytotoxicity assay (Promega, G1781) according to the manufacturer’s instructions. Absorbance values of wells containing effector cells alone and target cells alone were detected and subtracted as the background from the values of the co-cultures. Wells containing target cells alone were mixed with a lysis reagent for 30 min at 37 °C and the resulting luminescence was set as 100% lysis. Cytotoxicity was calculated by using the following formula: %Cytotoxicity = (Experimental − Effector spontaneous − Target spontaneous)/(Target maximum − Target spontaneous) × 100%

### Xenogenic mouse models

We used NSG (NOD-Prkdc^scid^IL2rg^tm1^/Bcgen, Beijing Biocytogen Co., Ltd) mouse model to assess the in vivo antitumor effect of transduced CAR-T cells. All mouse experiments were strictly followed the ethical regulations and approved by Institutional Animal Care and Use Committee of Sun Yat-Sen University. To observe antitumor activity of CAR-T cells in vivo, 6–8 weeks old female NSG mice were inoculated subcutaneously (s.c.) into the right flank with 5 × 10^6^ SKOV3/luc cells in 100 μL of 50:50 Matrigel (Corning) and phosphate-buffered saline (PBS). On day 7, the mice were intraperitoneally (i.p.) infused with VivoGlo Luciferin (Promega, 150 mg kg^−1^ body weight), anesthetized with isoflurane, and imaged on an in vivo imaging system (IVIS) with Living Image software (PerkinElmer). Then 1 × 10^6^ transduced CAR-T cells (in 200 μl of PBS) were adoptively transferred into tumor-bearing mice via the tail vein and all mice were i.p. injected IL-2 at 1 μg per mouse every 3 days. For re-challenge experiment, 2 × 10^6^ transduced CAR-T cells (in 200 μl of PBS), which was double the dose than that for the above experiment, were adoptively transferred to ensure tumors can be eradicated in conventional Her2-CAR-T cell-treated group. Mice were re-analyzed on the IVIS once a week. No randomization was used.

For other animal experiments, 5 × 10^6^ SKOV3 cells or 2 × 10^6^ Raji cells suspended in 0.1 ml of PBS were injected s.c. into the right flank of 6–8 weeks old female mice. CAR-T cells were infused via the tail vein 7 days later and volume of tumors was measured by two-dimensional measurements (mm): Volume of tumor = length × width^2^/2. Mice were i.p. injected IL-2 at 1 μg per mouse every 3 days. Mice were killed when the mean tumor burden in the control mice reached 1500–2000 mm^3^. Some tissues were digested with collagenase type IV (2 mg ml^−1^, Sigma) at 37 °C for 30 min and tumor-infiltrating T cells were separated by centrifugation on a discontinuous Percoll gradient (Haoyang, China). Others were used for immunohistochemistry (IHC) and immunofluorescence assays. For animal experiment-involved checkpoint blockade, anti-PD-1 blocking antibody (clone EH12.2H7, 329946, Biolegend) was i.p. injected at 200 μg per mouse every 3 days.

### Genome-wide transcriptional profiling

For microarray analysis, the purified RNA from tumor-infiltrating T cells in Trizol was shipped on dry ice to Aksomics (Shanghai, China) for analysis via Agilent Whole Human genome Oligo Microarray platform. The RNA preparation and microarray hybridization were performed according to the manufacturer’s instructions. Briefly, total RNA from each sample was amplified and transcribed into fluorescent cRNA using the manufacturer’s instructions. The labeled cRNAs were hybridized onto the Whole Human Genome Oligo Microarray (4 × 44 K, Agilent Technologies). After washing the slides, the arrays were scanned by the Agilent Scanner G2505C. Agilent Feature Extraction software (version 11.0.1.1) was used to analyze the acquired array images. Quantile normalization and subsequent data processing were performed using the GeneSpring GX v12.1 software package (Agilent Technologies). After quantile normalization of the raw data, differentially expressed genes were identified through fold-change filtering. Through *z*-score normalization, the transcriptional profiles data of differentially expressed genes were used for visualization by heatmap with MEV software (http://www.tm4.org/). GO enrichment analysis was applied to determine the roles of these differentially expressed genes enriched in specific GO biological process terms.

### ATAC-seq

ATAC-seq was performed with two independent experiments per condition^60^. Briefly, nuclei were isolated from 50,000 sorted cells per replicate using a solution of 10 mM Tris-HCl, 10 mM NaCl, 3 mM MgCl_2_, and 0.1% IGEPAL CA-630. Immediately following nuclei isolation, the transposition reaction was conducted using Nextera Tn5 transposase and TD buffer (Illumina) for 45 min at 37 °C. Transposed DNA fragments were purified using a Qiagen MinElute Kit, barcoded with dual indexes (Illumina Nextera), and PCR amplified using NEBNext High Fidelity 2 × PCR master mix (New England Labs). The size distribution and molarity of the sequencing library were determined by using an Agilent Bioanalyzer and KAPA quantitative RT-PCR (KAPA Biosystems). Sequencing was performed using an Illumina HiSeq X ten platform to acquire at least ∼ 50 M fragments per sample. Paired-end reads were mapped to the hg38 reference genome using Bowtie2. Only concordantly mapped pairs were kept for further analysis. Peak calling was performed using MACS v1.4 to identify areas of sequence tag enrichment. These peaks were displayed in the IGV genome browser and further processed for annotation and for differential open chromatin detection.

### Tissue processing and immunohistochemistry

Tissue samples were fixed, processed, and stained according to standard procedures. Briefly, tumors were resected from the mice and fixed with neutrally buffered 4% formaldehyde and applied to hematoxylin and eosin (H&E) staining conducted by Biopathology Institute Co., Ltd (Servicebio, China). For IHC, resected tumor tissues were immersed in Tissue-Tek O.C.T. Compound (4583, Sakura Finetek) and snap-frozen to produce cryosections by freezing microtome (Thermo NX50). Primary antibodies used for IHC staining were polyclonal rabbit anti-human CD3 mAb (17617-1-AP, proteintech) or polyclonal rabbit anti-human IFN-γ mAb (ab9657, Abcam). Images of both H&E staining samples and IHC sections were obtained using the microscope (Leica, DM6000B).

### Immunofluorescence

Resected tumor tissues were immersed in Tissue-Tek O.C.T. Compound (4583, Sakura) and snap-frozen to produce cryosections. CAR-T cells were stained with Alexa Fluor 488-conjugated donkey anti-mouse IgG H&L (polyclonal, ab150109, Abcam). Primary antibodies were as follows: rabbit anti-human CD56 antibody (Clone EP2567Y, ab75813, Abcam) and goat anti-human IFN-γ antibody (AF-285-SP, R&D). Alexa Fluor 594-conjugated donkey anti-rabbit IgG H&L (ab150076, Abcam) and Alexa Fluor 647-conjugated donkey anti-goat IgG H&L (ab150075, Abcam) were used as secondary antibodies. In Situ Cell Death TMR (Roche BR, 12156792910) was used for labeling apoptosis tissue. DAPI (4′,6-diamidino-2-phenylindole; Thermo Fisher Scientific) was used for the staining of the nucleus. Fluorescent signals were detected using the laser scanning confocal microscope (ZEISS LSM 800). For the quantification of immunofluorescence results, the images of margins between the living and apoptosis-undergoing tumor tissue were further analyzed with Imaris software (Version 7.4) (BITPLANE) using the Spot function to locate and enumerate CAR-T cells, IFN-γ, or CD56-positive cells, based on size and intensity threshold. Alternatively, the absolute numbers of CD8^+^ T cells, IFN-γ, or CD56-positive cells spot per mm^2^ in nine high-power fields of interested areas were statistically analyzed (three separate fields from each mouse and three mice from each group).

### LIPSTIC assay

LIPSTIC assay was conducted by following previous report with minor modifications^44^. Briefly, at the 5′-end of CD56 cDNA truncated the signal peptides; *SrtA* gene or *G5* gene with signal peptides at the 5′-end was added respectively by PCR-based linking. G5-CD56mut (CD56 with mutations K303A and F305A) was used as control. They were connected with Her2-CAR through IRES motif, respectively. SrtA-PD-1 and G5-PD-L1 were also constructed by the same method as positive control. For in vitro assay, CD8^+^ T cells were transduced with recombinant lentiviruses carrying SrtA-CD56-Her2-CAR or G5-CD56-Her2-CAR/G5-CD56mut-Her2-CAR separately mixed at 1:1 ratio (10^6^ cells of each population) in a 1.5 ml conical tube and chemically synthesized AlexaFluor488-SELPETGG was added to a final concentration of 100 μM. Cells were incubated at room temperature for 30 min and washed three times before fluorescence-activated cell sorting (FACS) staining. For LIPSTIC in vivo experiment, 6–8 weeks old female NSG mice were inoculated s.c. into the right flank with 5 × 10^6^ SKOV3 cells in 100 μL of 50:50 Matrigel (Corning) and PBS. G5-CD56-Her2-CAR-T/G5-CD56mut-Her2-CAR-T cells were labeled with carboxyfluorescein diacetate succinimidyl ester (CFSE). SrtA-CD56-Her2-CAR-T cells and G5-CD56-Her2-CAR-T/G5-CD56mut-Her2-CAR-T cells were mixed at 1:1 ratio (10^6^ cells per mouse in total) and then transferred to mice via the tail vein on day 7. Then biotin-aminohexanoic acid-LPETG was injected (i.p.) into mice 72 h later. After 12 h, mice were killed and tumor tissues were resected, followed by digestion for FACS analysis. SELPETGG (C-terminal amide, 95% purity) conjugated with AlexaFluor488 succinimidyl ester dye and biotin-aminohexanoic acid-LPETGS (C-terminal amide, 95% purity) were custom synthesized at Sinobioway (Zhangzhou, China).

### FRET assay

FRET assay was conducted by following previous procedures with minor modifications^61,62^. Briefly, at the 5′-end of CD56, CD56mut, or PD-1, cyan fluorescent protein (CFP) or yellow fluorescent protein (YFP) were added as indicated (at the 5′-end of CD56 cDNA truncated the signal peptides, CFP or YFP with signal peptides at the 5′-end was added respectively by PCR-based linking). The CD8^+^ T cells were transduced with recombinant lentiviruses carrying CFP-CD56, CD56mut, YFP-CD56, or YFP-PD-1 as indicated, which were mixed at 1:1 ratio (10^6^ cells of each population) and resuspended in phenol red-free medium and seeded in 96-well black plates (Corning). P3DE and P3G peptides at various concentrations were introduced to block the CD56-CD56 interaction. After incubation for 4 h, the emission at 495~505 nm (donor) and 500~550 nm (acceptor) were measured using the Glomax Discover detection system (Promega). Data were plotted as 500~550 nm/495~505 nm ratios vs. the concentration of P3DE/P3G peptides. Experiments are independently repeated at least three times and results are expressed as mean ± SEM from a single experiment performed in triplicate.

### Apoptosis assay

An Annexin V-FITC Apoptosis Detection Kit (DOJINDO, Shanghai, China) was used for detecting apoptosis according to the instructions of the manufacturer. After cultured with recombinant TNF-α (10 ng ml^−1^) and FasL (10 ng ml^−1^) for 24 h or cultured for 6 days in the deprivation of any extra cytokines, the CAR-T cells were labeled by anti-human APC-CD56 (Clone TULY56, 17-0566-42), Annexin V, and propidium iodide, and then detected by a flow cytometer BD FACS Aria.

### Statistical analysis

Statistical tests were conducted using Prism (GraphPad) software. All experimental data are presented as mean ± SEM. Gaussian distribution was confirmed by the Kolmogorov–Smirnov normality test. Unpaired, two-tailed Student’s *t*-tests and one-way analysis of variance with Tukey’s post hoc tests to further multiple comparisons were used.

### Reporting summary

Further information on research design is available in the Nature Research Reporting Summary linked to this article.

## Supplementary information

Supplementary Information

Reporting Summary

## Data Availability

All relevant data are included in this manuscript and/or in its Supplementary Information files. The microarray data have been deposited in GEO (Gene Expression Omnibus, https://www.ncbi.nlm.nih.gov/geo) with accession number GSE131107. ATAC-seq data have been deposited in SRA (Sequence Read Archive, https://trace.ncbi.nlm.nih.gov/Traces/sra) with accession number PRJNA542504. The source data underlying Figs. 1b–f, 2d, 3b, c, e, g, 4a, b, d, h, 5a–d, h, and 6a, b, and Supplementary Figs. 1d, e, 2b–d, 3a–c, 5a–c, 7a, b, 9, 11, and 12b, c are provided as a Source Data file.
