# Supplementary material for: Engineered triple inhibitory receptor resistance improves anti-tumor CAR-T cell performance via CD56
Source: Nat Commun. 2019 Sep 11;10:4109. doi: 10.1038/s41467-019-11893-4 (PMC6739330; doi:10.1038/s41467-019-11893-4)
Supplement: Supplementary file 1 — Supplementary Information [file 41467_2019_11893_MOESM1_ESM.pdf]

# Engineered Triple Inhibitory Receptor Resistance Improves Anti-Tumor Performance of CAR-T Cell via CD56 High Expression

**Zou F., et al.**

**This PDF file includes:**

Supplementary Figure 1-14

Supplementary Tables 1-2

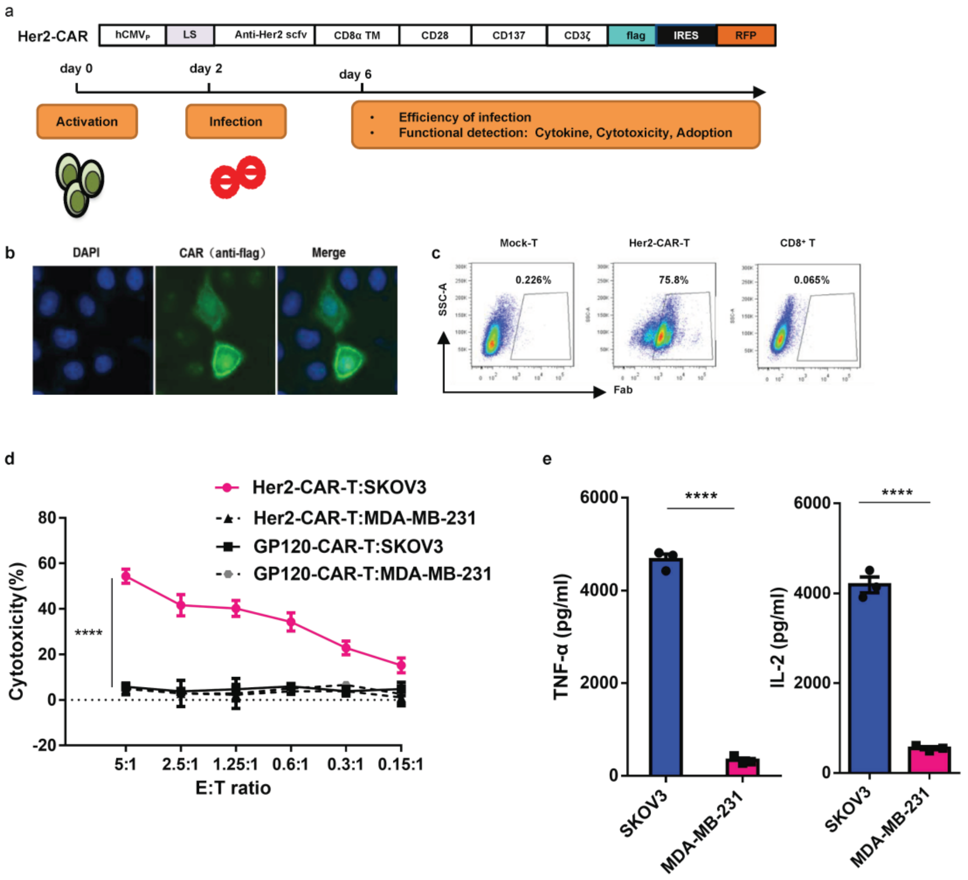


**Supplementary Figure 1. Generation of Her2-specific CAR-T cells. (a)** Top, schematic representation of the lentiviral vector carrying Her2-specific chimeric antigen receptor (Her2-CAR). Bottom, timeline of the transduction of CAR moiety into primary T cells by lentiviral infection. **(b)** Immunofluorescence staining for Her 2-CAR in HEK293T cells. **(c)** Representative CAR flow plots of T cells transduced with lentiviral particles by detecting the scFv fragment of CAR moiety with anti-Fab antibody. CD8$^{+}$ T cells transduced with empty vector served as mock-T cells. **(d)** Cytotoxic activity of Her2 CAR-T cells on SKOV3 targets cells were detected by LDH assay, and GP120-CAR-T cells were used as negative control (*n* = 3, 3 healthy donors). **(e)** TNF-α and IL-2 release after Her2-CAR-T cells co-cultured with target cells at E: T=5:1 (*n* = 3, 3 healthy donors).

****$P < 0.0001$ (one-way ANOVA with Tukey's post hoc tests (**d**); two-tailed Student's t-test (**e**)). All data are means ± s.e.m. Data shown are representative data from three independent experiments. The source data underlying **d** and **e** are provided as a Source Data file.

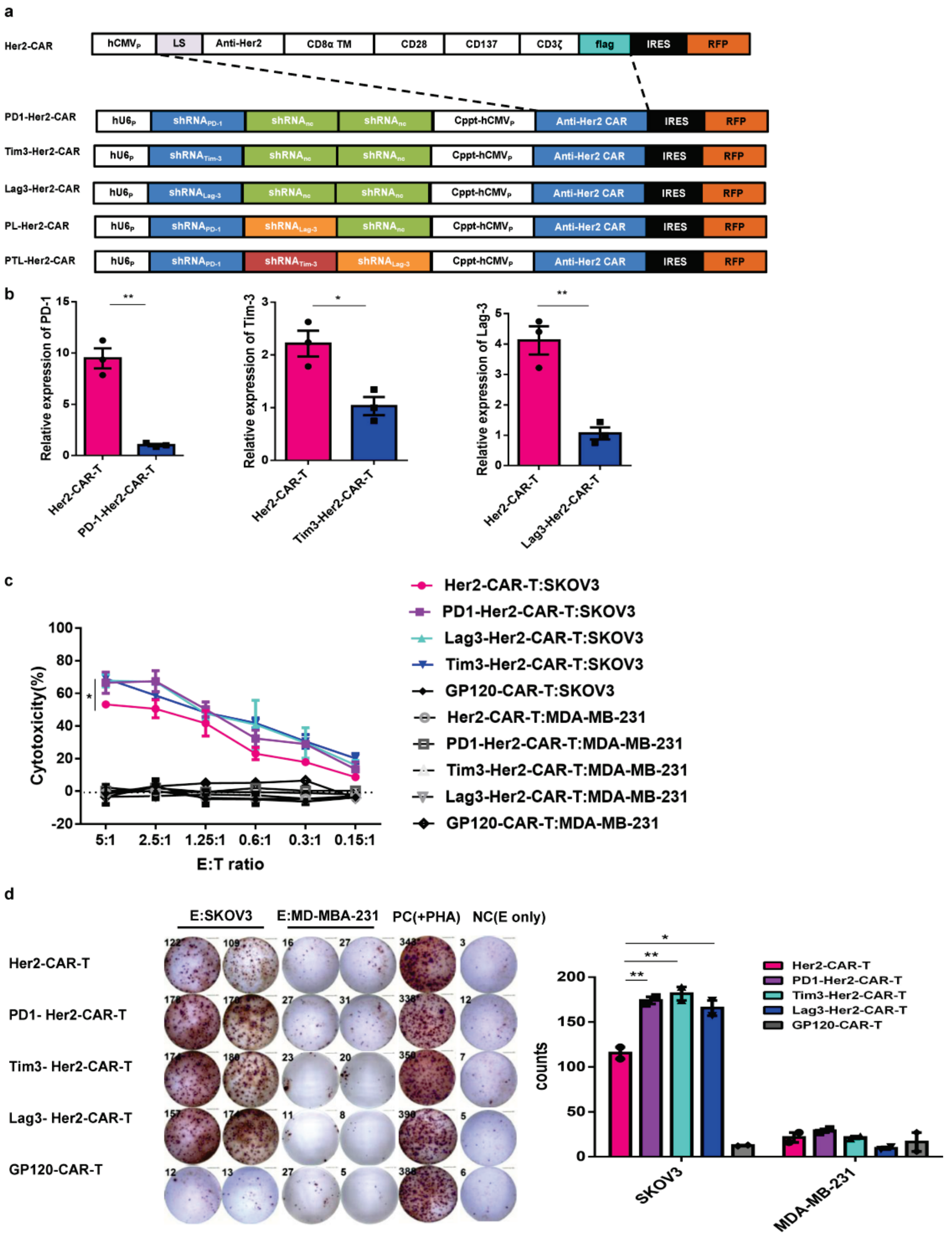


**Supplementary Figure 2. Her2-specific CAR-T cells carrying various co-receptor-specific shRNAs enhanced their capabilities to kill the tumor cells. (a)** Schematic representation of the lentiviral vectors carrying a Her-2-specific CAR moieties and one of sh-PD-1, sh-Lag-3, sh-Tim-3 or a combination of sh-PD-1 and sh-Lag-3, or a combination of sh-PD-1, sh-Lag-3, and sh-Tim-3. The second structure of those shRNA clusters was derived from the miR-106b cluster. **(b)** Relative mRNA

expression level of inhibitory receptors in Her2-CAR-T cells with down-regulation of PD-1, Lag-3, or Tim-3 respectively (14 days post infection) ($n$ = 3, 3 healthy donors). **(c)** Cytotoxic activity of PD-1-Her2-CAR, Tim-3-Her2-CAR, Lag-3-Her2-CAR-T cells (14 days post infection) on SKOV3 cells were detected by LDH assay, and GP120-CAR-T cells were used as mock CAR-T cells. **(d)** Immune response was evaluated by measuring the number of IFN-γ producing and spot-forming cells (SFCs) per $10^4$ CAR-T cells (14 days post infection) ($n$ = 2, 2 healthy donors). All the CAR-T cells added with PHA were used as positive control and CAR-T cells alone as negative control.

*$P$ < 0.05, **$P$ < 0.01 (one-way ANOVA with Tukey's post hoc tests (**c, d**); two-tailed Student's t-test (**b**)). All data are means ± s.e.m. Data shown are representative data from three independent experiments. The source data underlying **b-d** are provided as a Source Data file.

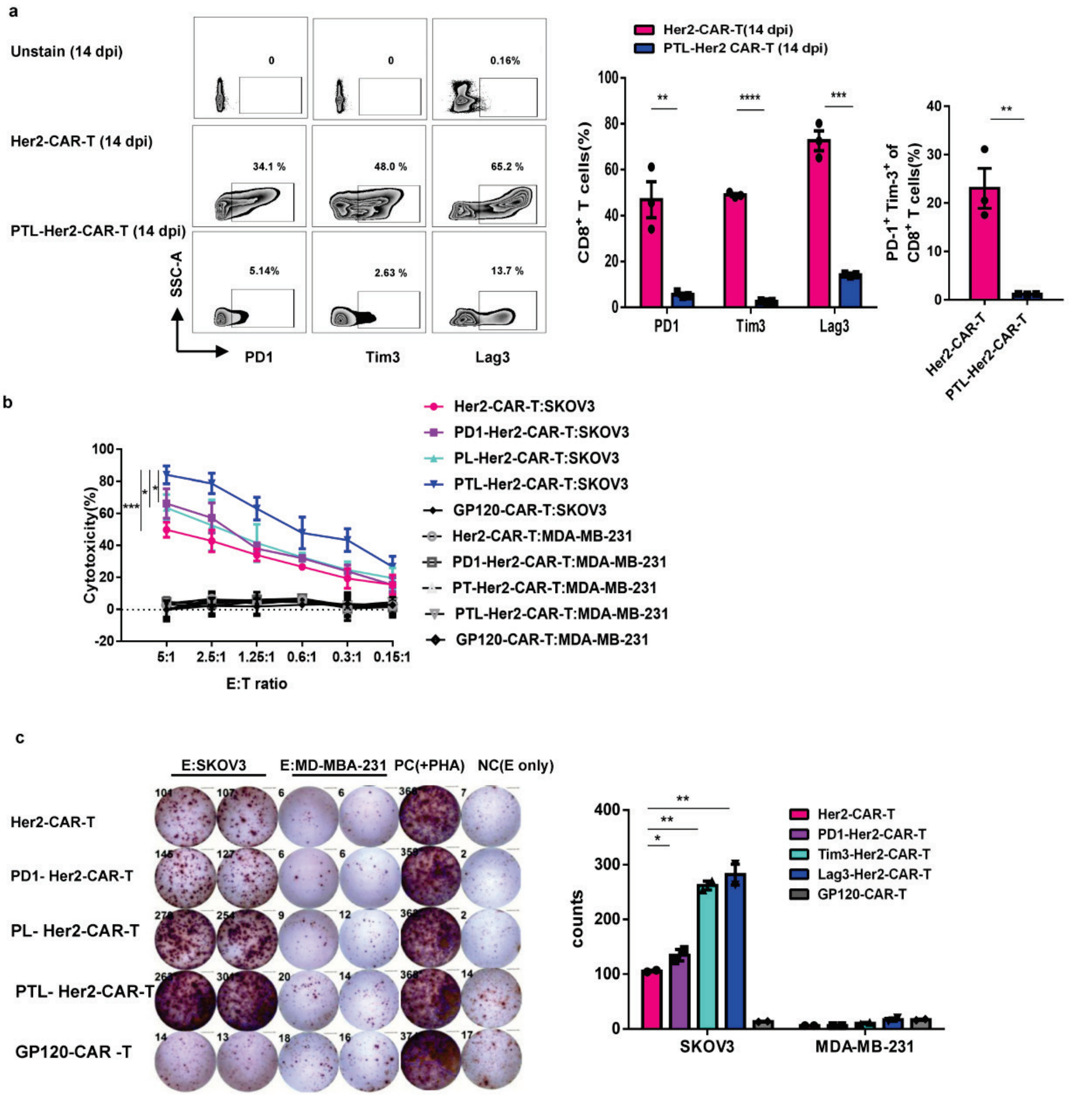


**Supplementary Figure 3. PTL-Her2-CAR-T cells exerted a potent capability to specifically kill the Her2-positive tumor cells. (a)** Left panel shows representative flow plots of inhibitory receptors on Her2-CAR-T cells and PTL-Her2-CAR-T cells 14 days post infection (14 dpi). Middle panel shows percentage of PD-1, Lag-3 or Tim-3 respectively positive cells gated as in left panel. Right panel shows percentage of PD-1 and Tim-3 double positive cells gated as in left panel (*n* = 3, 3 healthy donors). **(b)** Cytotoxic activities of Her2-CAR-T cells, PL-Her2-CAR-T cells, PTL-Her2-CAR-T cells on SKOV3/luc targets cells (*n* = 3, 3 healthy donors). **(c)**

Immune response was evaluated by measuring the IFN-γ-producing and spot-forming cells (SFCs) per $10^4$ CAR-T cells. All the CAR-T cells added with PHA were used as positive control and CAR-T cells alone as negative control. All the CAR-T cells used above were *in vitro* cultured for 14 days ($n$ = 2, 2 healthy donors).

*$P$ < 0.05, **$P$ < 0.01, ***$P$ < 0.001, ****$P$ < 0.0001 (one-way ANOVA with Tukey's post hoc tests (**b, c**); two-tailed Student's t-test (**a**)). All data are means ± s.e.m. Data shown are representative data from three independent experiments. The source data underlying **a-c** are provided as a Source Data file.

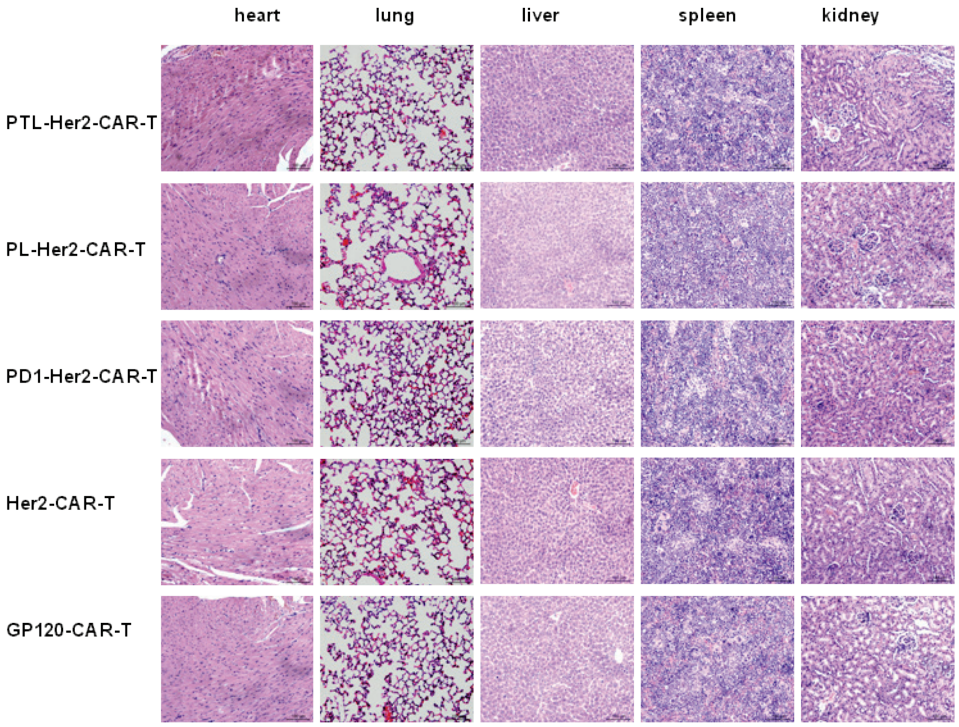


**Supplementary Figure 4. Her2-specific CAR-T cells with shRNAs for different co-receptors did not exhibit any toxicity on various normal tissues.**

Mice were euthanized 28 days after CAR-T cells infusion to measure their possible toxicity in heart, lung, liver, spleen and kidney tissues. Tissues were stained with haematoxylin and eosin staining. Scale bar, 100 µm. Data shown are representative data from two independent experiments.

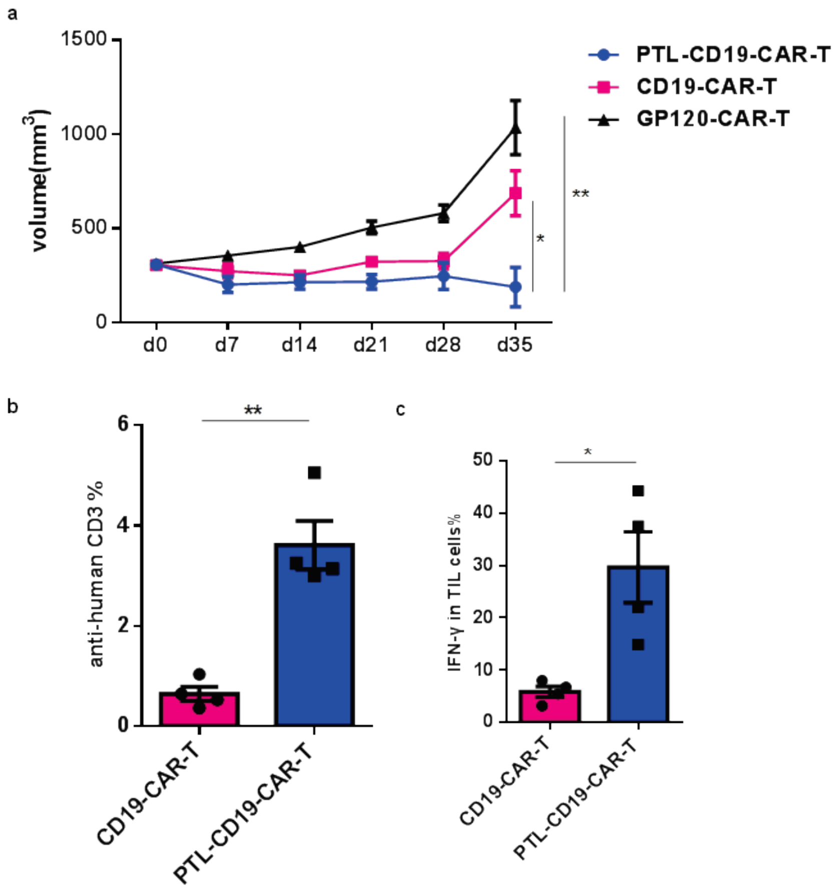


**Supplementary Figure 5. CD19-specific-CAR-T cells carrying shRNAs for PD-1, Lag-3, and Tim-3 (PTL-CD19-CAR-T cells) potently enhanced their capabilities to inhibit the growth of CD19-positive tumor cells.** $CD19^+$ Raji-bearing NSG mice in each group were treated with $1\times10^6$ CAR-T cells. At 28 days after CAR-T cells infusion, mice were euthanized and tumors were collected ($n$ = 3 for GP120-CAR-T cells treated group and $n$ = 4 for other groups). CAR-T cells were analyzed with flow cytometry. **(a)** Tumors' volume of mice. **(b)** Percentage of CAR-T cells in tumor. **(c)** Percentages of IFN-γ positive cells in tumor infiltrating CAR-T cells.

**P* < 0.05, ***P* < 0.01, (one-way ANOVA with Tukey's post hoc tests (**a**); two-tailed Student's t-test (**b, c**)). All data are means ± s.e.m. Data shown are representative data from two independent experiments. The source data underlying **a-c** are provided as a

Source Data file.

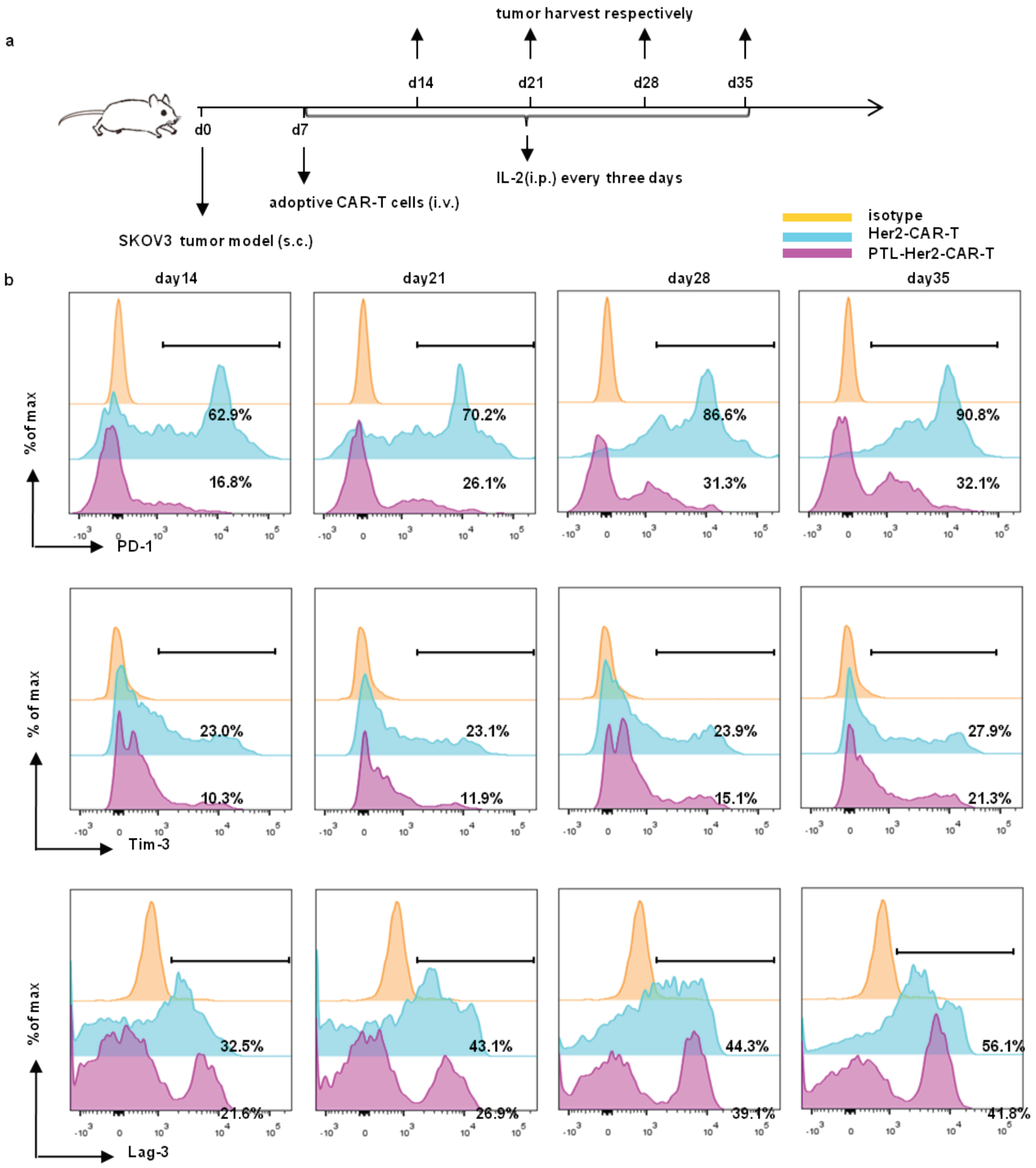


**Supplementary Figure 6. PTL-Her2-CAR T cells exerted persistent down-regulation of PD-1, Tim-3, and Lag-3 at different time points *in vivo*. (a)** Schematic diagram of a complete animal experiment. SKOV3/luc cells were inoculated subcutaneously into NSG mice. Seven days later, various CAR-T cells were infused via tail injection (1 × $10^6$ per mouse). Quarters of the mice were euthanized every 7 days since day 14 and tumor tissues were resected, followed by digestion for FACS analysis. **(b)** Representative flow cytometry analysis of inhibitory

receptors expression of CAR-T cells at different time points (gated on $CD8^+$ $Fab^+$ T cells). Data shown are representative data from two independent experiments.

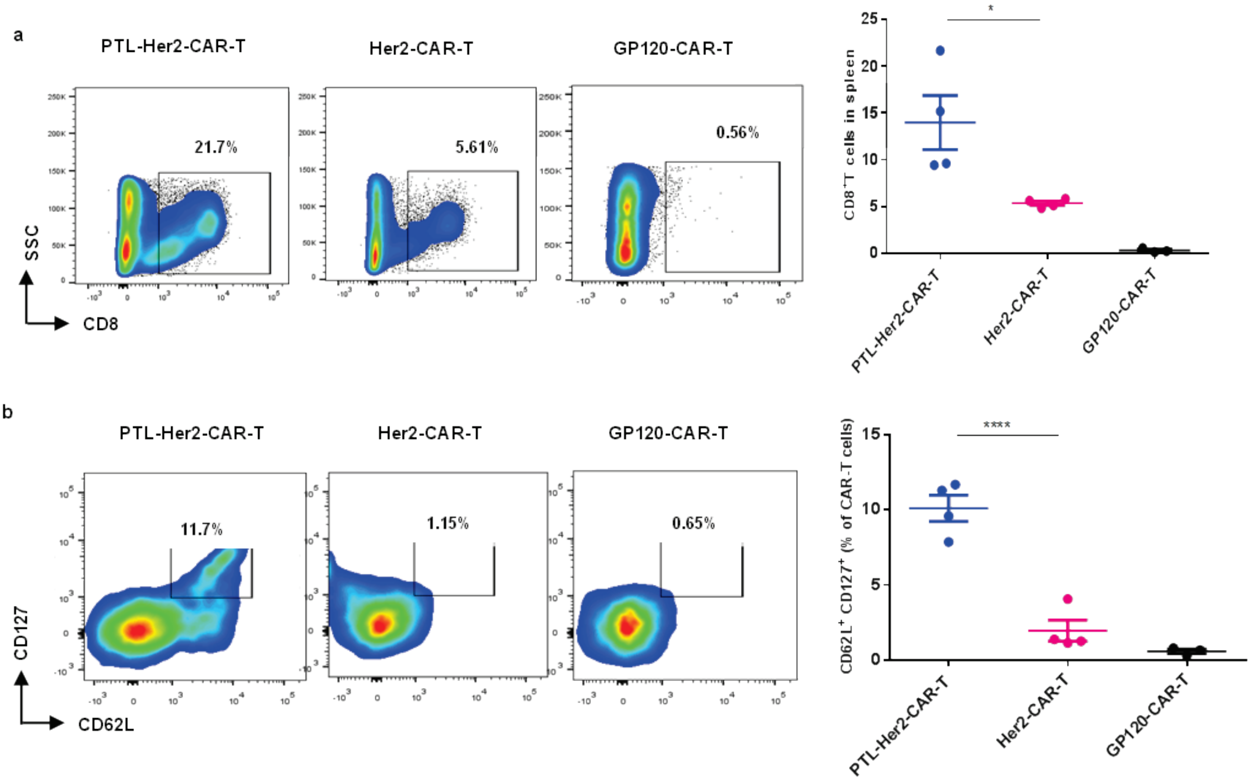


**Supplementary Figure 7. PTL-Her2-CAR-T cells developed memory phenotype.** SKOV3-bearing NSG mice in each group were treated with various CAR-T cells. At 60 days after CAR-T cells infusion, mice were euthanized and spleens were collected ($n$ = 3, GP120-CAR-T cells treated group; $n$ = 4, other groups). CAR-T cells were analyzed with flow cytometry. **(a)** Left panel, the representative flow plots of the recipient CAR-T cells identified as human $CD8^+$ T cells in spleen; right panel, the percentages of $CD8^+$ cells among different groups. **(b)** Left panel, the representative flow plots of the expression of human CD62L and CD127 (gated on $CD8^+$ T cells) in spleen; right panel, the percentages of CD62L and CD127 positive cells among different groups.

**$P < 0.01$, ****$P < 0.0001$ (one-way ANOVA with Tukey's post hoc tests (**a, b**)). All data are means ± s.e.m. Data shown are representative data from two independent experiments. The source data underlying **a** and **b** are provided as a Source Data file.

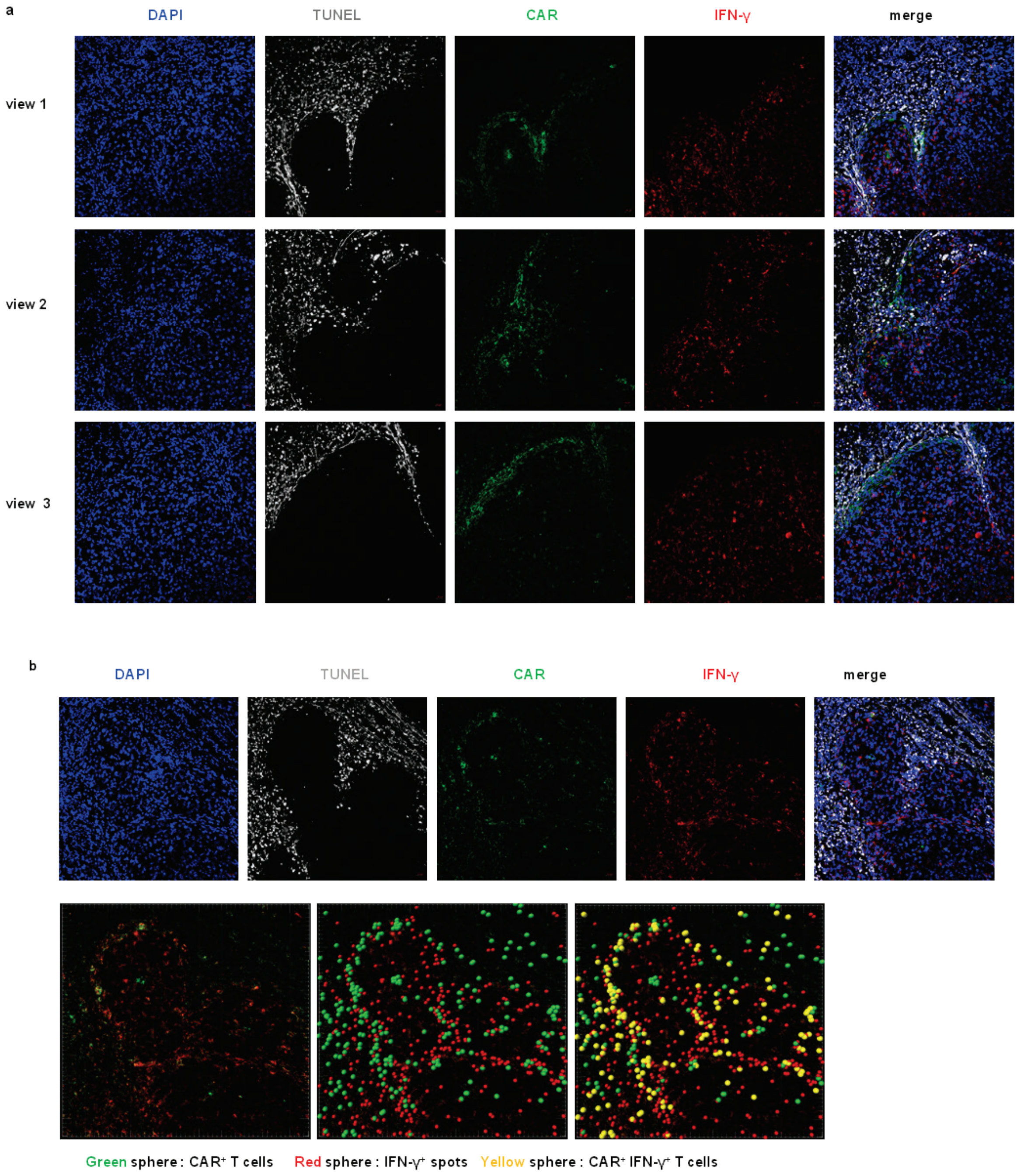


**Supplementary Figure 8. More views for immunofluorescence staining of tumor frozen tissue sections treated with PTL-Her2-CAR-T cells (extended pictures for Fig. 2c top panel) and an example of quantification diagram of immunofluorescence. (a)** CAR-T cells were stained with anti-mouse IgG H&L (Alexa Fluor 488), and IFN-γ was stained with anti-human IFN-γ (Alexa-Fluor647). Apoptosis-undergoing cells were stained by TUNEL assay, and nuclei were stained

with DAPI (blue). Scale bar, 20 μm. Pictures shown are representative of two independent experiments. (**b**) Up panel, one example immunofluorescence image from Fig. 2c. Down panel, the Spot function of Imaris software located and enumerated CAR-T cells (green), IFN-γ spots (red) and $CAR^+$ IFN-$\gamma^+$ T cells (yellow) based on size and intensity threshold.

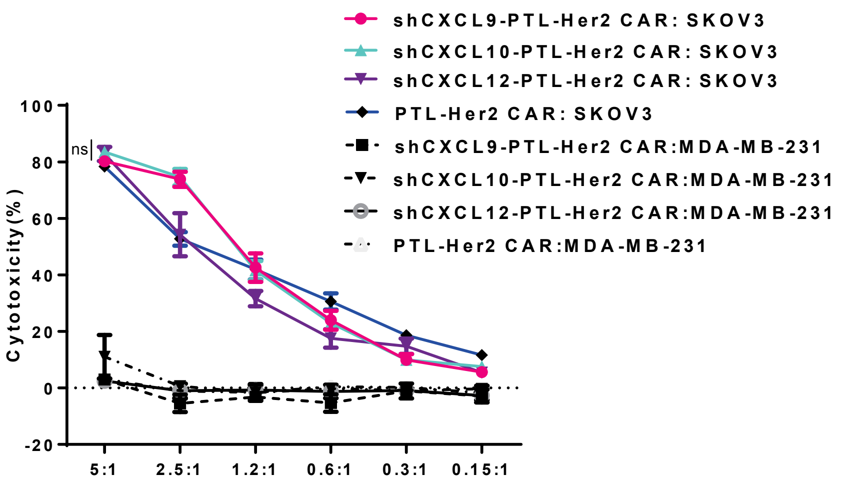


**Supplementary Figure 9. Chemokine ligand knockdown by shRNA did not reduce the specific cytotoxicity of PTL-Her2-CAR-T cells.** Cytotoxic activities of PTL-Her2-CAR-T cells carrying shRNAs for CXCL9, CXCL10, CXCL12 respectively to SKOV3 cells were detected by LDH assay (*n* = 3, 3 healthy donors). ns, no significant difference (one-way ANOVA with Tukey's post hoc tests). All data are means ± s.e.m. Data shown are representative data from three independent experiments. The source data underlying this figure is provided as a Source Data file.

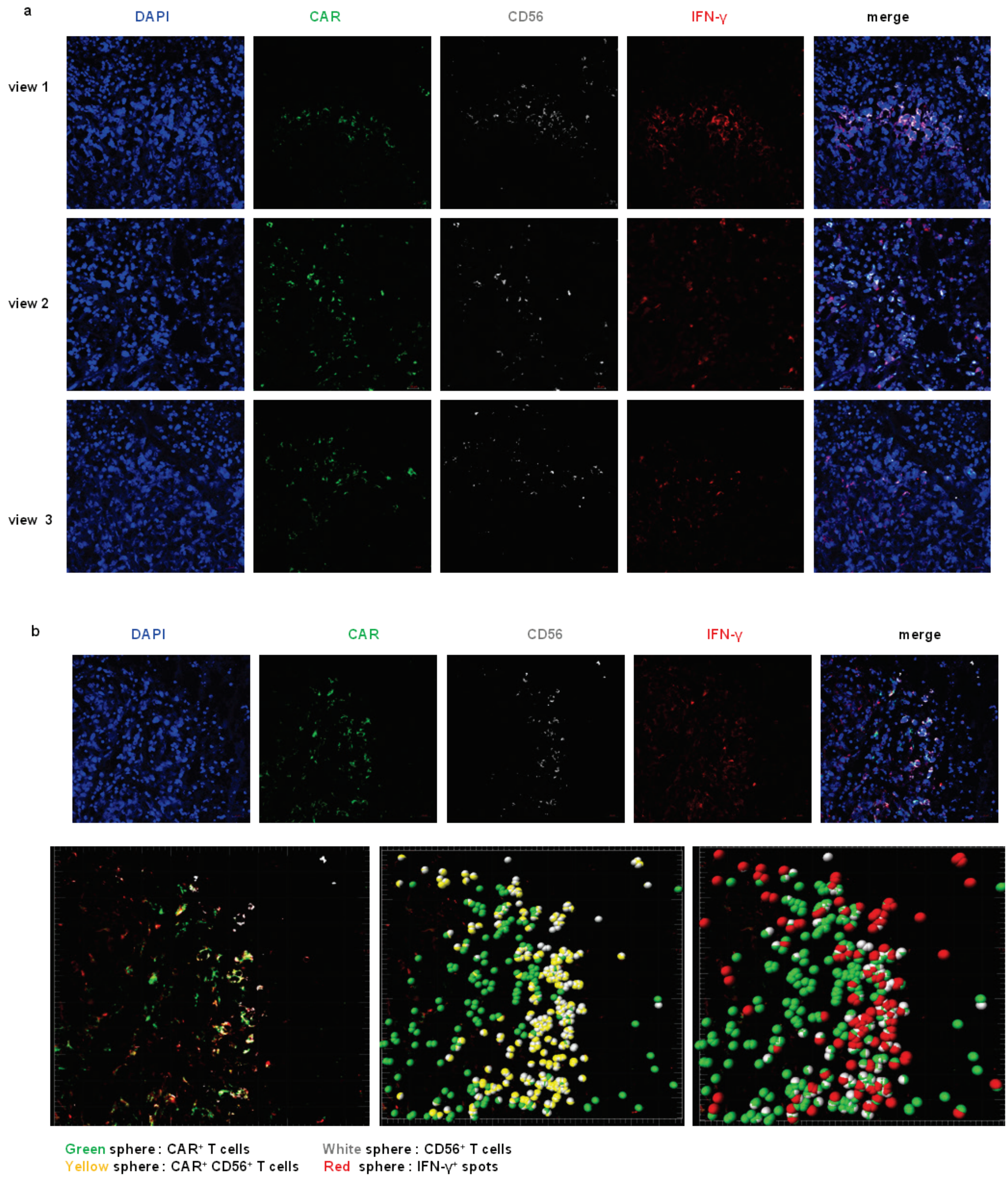


**Supplementary Figure. 10. More views for immunofluorescence staining of frozen tissue sections of tumors treated with PTL-Her2-CAR-T cells (extended pictures for Fig. 3f top panel) an example of quantification diagram of immunofluorescence. (a)** CAR-T cells were stained with anti-mouse IgG H&L (Alexa Fluor 488). CD56 was stained with anti-human CD56 (Alexa Fluor 594) and

IFN-γ was stained with anti-human IFN-γ (Alexa Fluor 647). Nuclei were stained with DAPI (blue). Scale bar, 20 μm. Pictures shown are representative of two independent experiments. (**b**) Up panel, one example of immunofluorescence image from the PTL-Her2-CAR-T group in Fig. 3f. Down panel, the Spot function of Imaris software located and enumerated CAR-T cells (green), $CD56^+$ T cells (white) , $CAR^+$ $CD56^+$ T cells (yellow) and IFN-γ spots (red), based on size and intensity threshold.

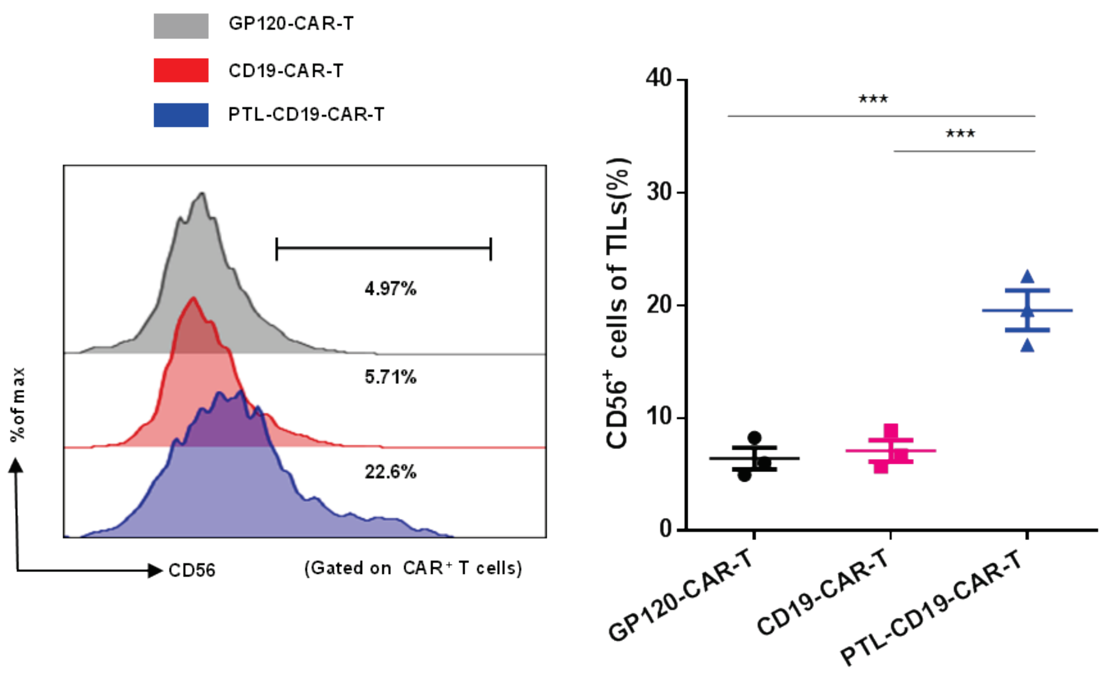


**Supplementary Figure 11. CD56 was highly expressed on CD19$^{+}$ tumor infiltrated-PTL-CD19-CAR-T cells.** The CD19$^{+}$ Raji cells were inoculated subcutaneously into NSG mice. Seven days later, Raji-bearing mice were treated with various CAR-T cells (*n* = 3 for each group). At day 28 after CAR-T cell infusion, mice were euthanized and tumors were collected. CAR-T cells were analyzed with flow cytometry.

****P* < 0.001(one-way ANOVA with Tukey's post hoc tests). All data are means ± s.e.m. Data shown are representative data from two independent experiments. The source data underlying this figure is provided as a Source Data file.

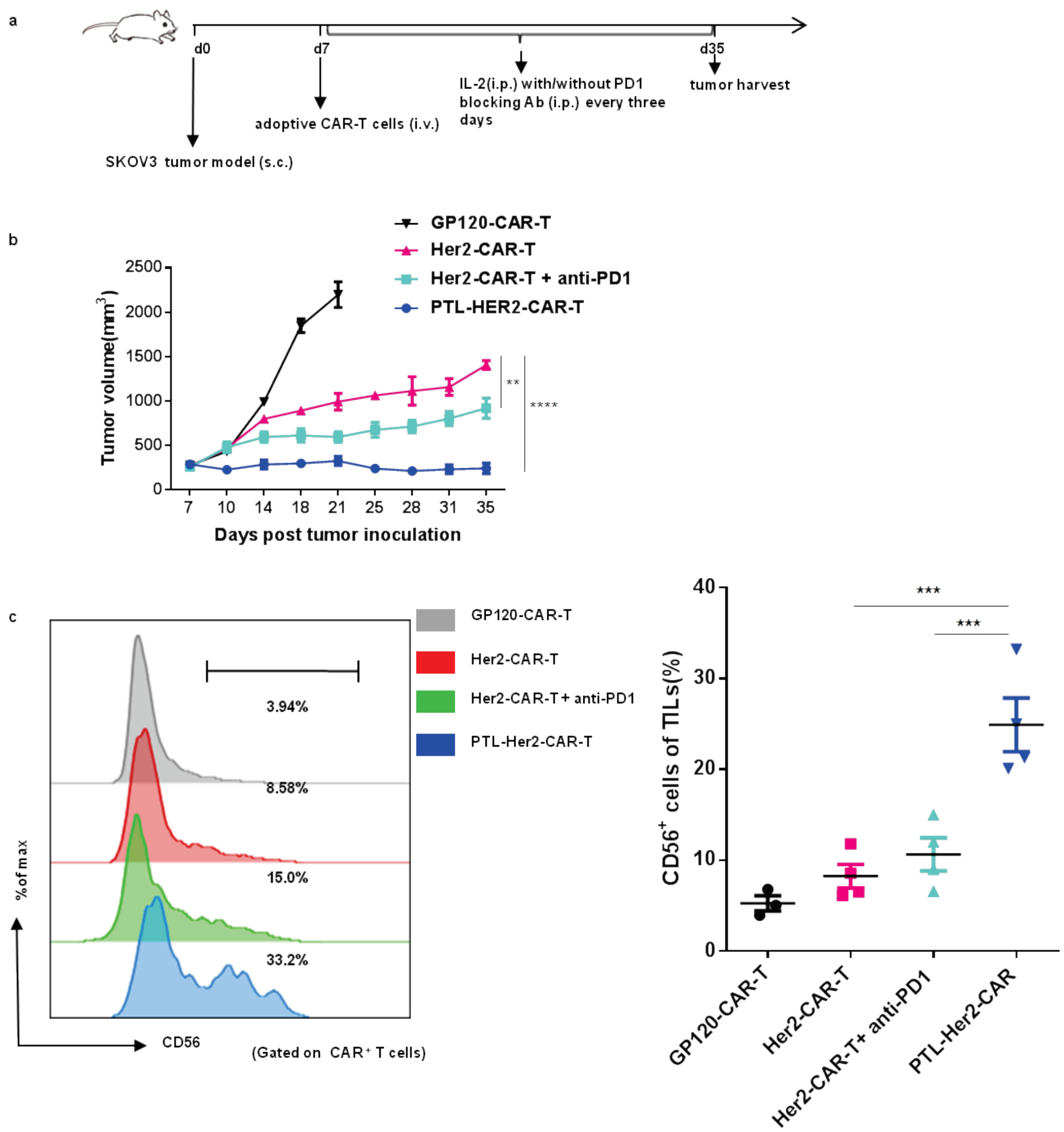


**Supplementary Figure 12. Her-2-CAR-T cells with anti-PD-1mAbtreated mice also showed an enhanced anti-tumor phenotype but not significant increase of CD56. (a)** Schematic diagram of a complete animal experiment. SKOV3 cells were inoculated subcutaneously into NSG mice. Seven days later, SKOV3-bearing mice were treated with low dose various CAR-T cells ($5 \times 10^5$ per mouse) with or without anti-PD-1 blocking antibody at 200 μg per mouse every 3 days ($n = 3$, GP120-CAR-T cells treated group; $n = 4$, other groups). At day 28 after CAR-T cells infusion, mice were euthanized and tumors were collected. CAR-T cells were analyzed with flow

cytometry. **(b)** Tumors' volume of mice. **(c)** Left panel shows representative flow cytometry analysis of CD56 positive CAR-T cells; right panel shows the percentages of CD56 positive cells on tumor infiltrating CAR-T cells among different groups (gated on $Fab^+$ CAR-T cells).

$**P < 0.01$, $***P < 0.001$, $****P < 0.0001$ (one-way ANOVA with Tukey's post hoc tests (**b**,**c**)). All data are means ± s.e.m. Data shown are representative data from two independent experiments. The source data underlying **b** and **c** are provided as a Source Data file.

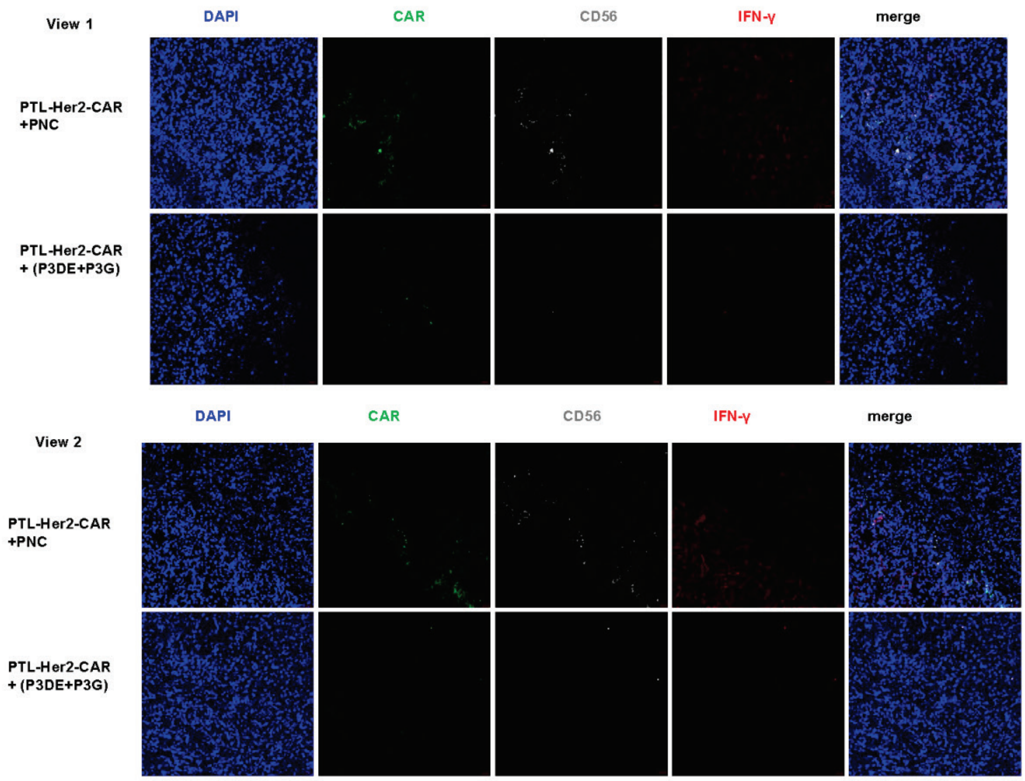


**Supplementary Figure 13. More views for immunofluorescence staining of frozen tissue sections of xenograft tumors treated with PTL-Her2-CAR-T cells in the presence of P3DE + P3G peptides or PNC (extended pictures for Fig. 5f)**. CAR-T cells were stained with anti-mouse IgG H&L (Alexa Fluor 488). CD56 was stained with anti-human CD56 (Alexa Fluor 594), IFN-γ was stained with anti-human IFN-γ (Alexa Fluor 647), and nuclei were stained with DAPI (blue). Scale bar, 20 μm.

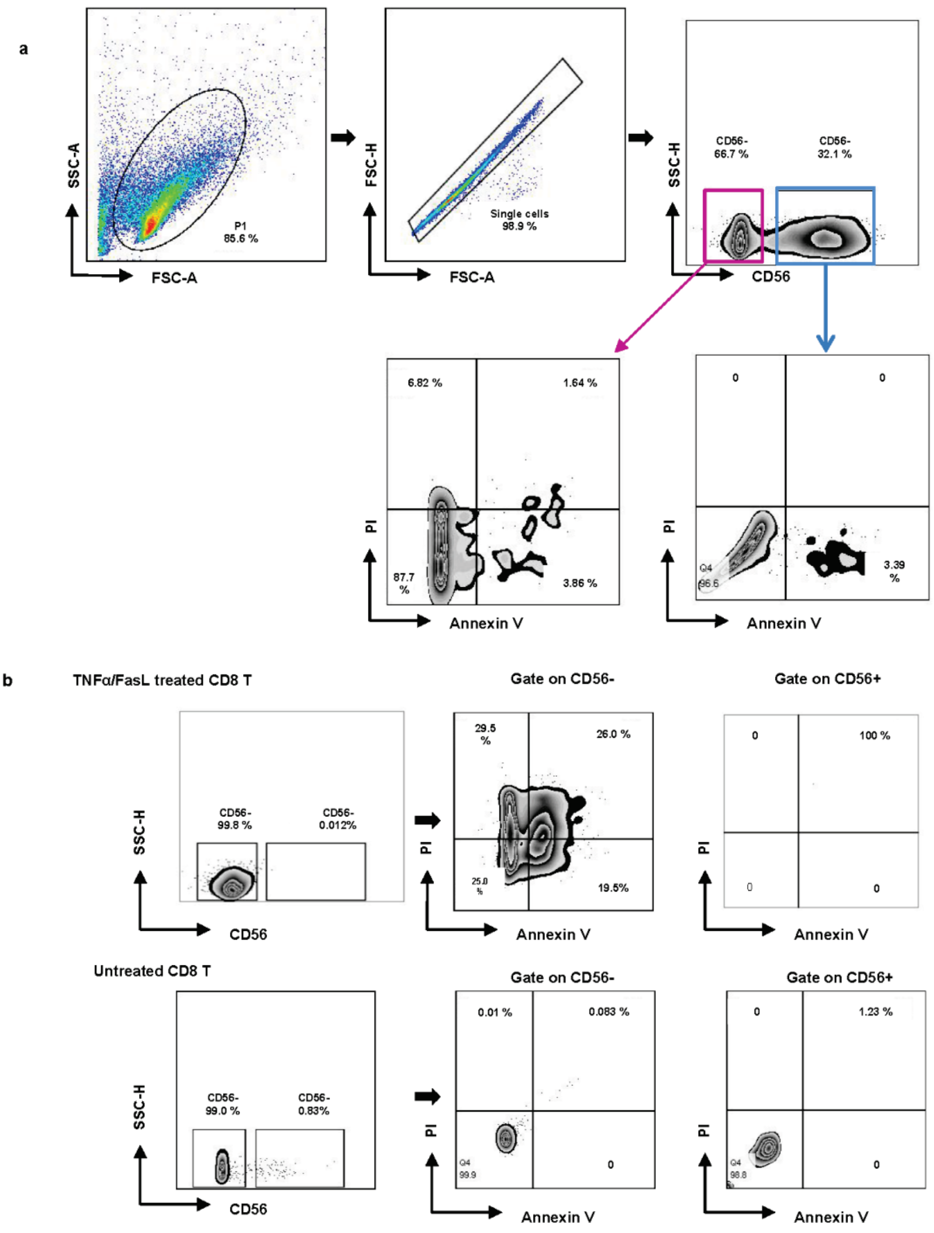


**Supplementary Figure 14. FACS gating strategy and experimental controls for Figure 6. (a)** FACS gating strategy for analyzing the apoptosis phenotype of $CD56^+$ or $CD56^-$ CAR-T cells separately in Figure 6. **(b)** Flow plots of apoptotic cells proportion in primary $CD8^+$ T cells co-cultured without (up panel, negative control) or with (down panel, positive control) the treatment of TNF-α and FasL (10 ng $ml^{-1}$).

**Supplementary Table 1.** Sequences of siRNAs.

| Target gene | Name in the text | Target sequence |
|---|---|---|
| PD-1 | sh-PD-1 | GCTTCGTGCTAAACTGGTA |
| Tim-3 | sh-Tim-3 | ACTCTAGATTGGCCAATGA |
| Lag-3 | sh-Lag-3 | TGGCGACTTTACCCTTCGA |

**Supplementary Table 2.** Sequences of primers for qRT–PCR.

| **primer name** | **sequence** |
|---|---|
| qRT-PCR-PD-1-F | CCAGGATGGTTCTTAGACTCCC |
| qRT-PCR-PD-1-R | TTTAGCACGAAGCTCTCCGAT |
| qRT-PCR-Tim-3-F | CTGCTGCTACTACTTACAAGGTC |
| qRT-PCR-Tim-3-R | GCAGGGCAGATAGGCATTCT |
| qRT-PCR-Lag-3-F | GCCTCCGACTGGGTCATTTT |
| qRT-PCR-Lag-3-R | CTTTCCGCTAAGTGGTGATGG |
| qRT-PCR-CXCL9-F | CCAGTAGTGAGAAAGGGTCGC |
| qRT-PCR-CXCL9-R | AGGGCTTGGGGCAAATTGTT |
| qRT-PCR-CXCL10-F | GTGGCATTCAAGGAGTACCTC |
| qRT-PCR-CXCL10-R | TGATGGCCTTCGATTCTGGATT |
| qRT-PCR-CXCL12-F | ATTCTCAACACTCCAAACTGTGC |
| qRT-PCR-CXCL12-R | ACTTTAGCTTCGGGTCAATGC |
| qRT-PCR-BCL2-F | GAACTGGGGGAGGATTGTGG |
| qRT-PCR-BCL2-R | ACAAAGGCATCCCAGCCTC |
| qRT-PCR-IFNG-F | CAGCTCTGCATCGTTTTGGG |
| qRT-PCR-IFNG-R | TCTGTCACTCTCCTCTTTCCA |
| qRT-PCR-TNFA-F | GCTGCACTTTGGAGTGATCG |
| qRT-PCR-TNFA-R | GAGGGTTTGCTACAACATGGG |
| qRT-PCR-CD56-F | GGCATTTACAAGTGTGTGGTTAC |
| qRT-PCR-CD56-R | TTGGCGCATTCTTGAACATGA |
| qRT-PCR-GAPDH-F | AGAAGGCTGGGGCTCATTTG |
| qRT-PCR-GAPDH-R | AGGGGCCATCCACAGTCTTC |
